# Meningococcal A conjugate vaccine coverage in the meningitis belt of Africa from 2010 to 2021: a modelling study

**DOI:** 10.1016/j.eclinm.2022.101797

**Published:** 2023-01-05

**Authors:** Rose G. Bender, Jasmine Shen, Aleksandr Aravkin, André Arsène Bita Fouda, Ado M. Bwaka, Natalie C. Galles, Emily Haeuser, Simon I. Hay, Anderson Latt, Jason M. Mwenda, Emma L.B. Rogowski, Alyssa N. Sbarra, Reed J.D. Sorensen, Avina Vongpradith, Claire Wright, Peng Zheng, Jonathan F. Mosser, Hmwe H. Kyu

**Affiliations:** a Institute for Health Metrics and Evaluation, University of Washington, Seattle, WA, USA; b Department of Health Metrics Sciences, University of Washington, Seattle, WA, USA; c School of Medicine, University of Washington, Seattle, WA, USA; d Department of Applied Mathematics, University of Washington, Seattle, WA, USA; e World Health Organization Regional Office for Africa, Brazzaville, Republic of Congo; f World Health Organization Regional Office for Africa, Inter-Country Support Team, Ouagadougou, Burkina Faso; g World Health Organization Regional Office for Africa, Emergency Preparedness and Response Cluster, Dakar Emergency Hub, Dakar, Senegal; h Department of Infectious Disease Epidemiology, London School of Hygiene and Tropical Medicine, London, UK; i Meningitis Research Foundation, Bristol, UK

**Keywords:** Meningitis, MenAfriVac, Meningococcal vaccination, Meningitis belt, Vaccination coverage, Meningococcal disease

## Abstract

**Background:**

As of the end of 2021, twenty-four countries in the African meningitis belt have rolled out mass campaigns of MenAfriVac®, a meningococcal A conjugate vaccine (MACV) first introduced in 2010. Twelve have completed introduction of MACV into routine immunisation (RI) schedules. Although select post-campaign coverage data are published, no study currently comprehensively estimates MACV coverage from both routine and campaign sources in the meningitis belt across age, country, and time.

**Methods:**

In this modelling study, we assembled campaign data from the twenty-four countries that had introduced any immunisation activity during or before the year 2021 (Benin, Burkina Faso, Burundi, Cameroon, Central African Republic, Chad, Côte d’Ivoire, Democratic Republic of the Congo, Ethiopia, Eritrea, the Gambia, Ghana, Guinea, Guinea Bissau, Kenya, Mali, Mauritania, Niger, Nigeria, Senegal, South Sudan, Sudan, Togo and Uganda) via WHO reports and RI data via systematic review. Next, we modelled RI coverage using Spatiotemporal Gaussian Process Regression. Then, we synthesized these estimates with campaign data into a cohort model, tracking coverage for each age cohort from age 1 to 29 years over time for each country.

**Findings:**

Coverage in high-risk locations amongst children aged 1–4 in 2021 was estimated to be highest in Togo with 96.0% (95% uncertainty interval [UI] 92.0–99.0), followed by Niger with 87.2% (95% UI 85.3–89.0) and Burkina Faso, with 86.4% (95% UI 85.1–87.6). These countries had high coverage values driven by an initial successful mass immunisation campaign, followed by a catch-up campaign, followed by introduction of RI. Due to the influence of older mass vaccination campaigns, coverage proportions skewed higher in the 1–29 age group than the 1–4 group, with a median coverage of 82.9% in 2021 in the broader age group compared to 45.6% in the narrower age group.

**Interpretation:**

These estimates highlight where gaps in immunisation remain and emphasise the need for broader efforts to strengthen RI systems. This methodological framework can be applied to estimate coverage for any vaccine that has been delivered in both routine and supplemental immunisation activities.

**Funding:**

10.13039/100000865Bill and Melinda Gates Foundation.

**Research in context**

**Evidence before this study**

Routine immunisation coverage data for meningococcal A conjugate vaccine (MACV) is available for select country-years on the WHO Immunization Data portal. Post-campaign coverage estimates are released by WHO in their Weekly Epidemiological Report. Bwaka et al. have previously collated this campaign data and published a manuscript on the status of the rollout of MACV; however, this estimate did not incorporate additional coverage provided by routine immunisation. We searched PubMed with the search terms meningococcal vaccines [MeSH] AND (“meningitis belt” OR “MenAfriVac” OR “Africa”) AND (“coverage” OR “uptake” OR “rollout”), with no language restrictions, for articles published from January 1, 2010, up to October 17, 2022. We did not identify any additional studies that provided comparable population-level MACV coverage estimates from both campaigns and routine immunisation in all meningitis belt countries across time.

**Added value of this study**

Synthesizing coverage data from both campaigns and routine immunisation, we produce comprehensive estimates of population-level MACV coverage in all meningitis belt countries where it has been administered routinely or as part of a MACV campaign, from 2010 to 2021. We provide these estimates for both high-risk areas of each country and for each country as a whole. This allows for comparisons of vaccine coverage across space and time and may help policymakers target areas for further vaccination activity.

**Implications of all the available evidence**

MACV rollout has been very successful, with high uptake and coverage levels that persist in the population over time. However, the absence in some locations of routine immunisation implementation following campaigns has led to gaps in coverage, which may put these countries at risk for *Neisseria meningitidis* serogroup A (MenA) resurgence. To achieve the WHO goal of eliminating meningitis by 2030, rapid and sustained implementation across the meningitis belt of routine immunisation with MenA containing conjugate vaccine is essential.

## Introduction

Meningococcal meningitis, infection of the meninges by *Neisseria meningitidis*, was responsible for an estimated 54,400 (95% uncertainty interval (UI): 17,600–107,000) deaths globally in 2019.^1^ Amongst survivors of meningococcal meningitis, 10–25% experience disabling sequelae.^2^ The meningitis belt, which reaches across the Sahel from the East to West coast of Sub-Saharan Africa, is high-burden area prone to epidemics in the dry season.^3^ Prior to vaccination campaigns, *N. meningitidis* serogroup A (MenA) was historically responsible for as many as 90% of all-age meningitis cases in the area.^4^ Within the meningitis belt, there is substantial epidemiological variation both between and within countries, with highest epidemic risk and disease burden found in Burkina Faso, Chad, Ethiopia, Mali, Niger, Nigeria, and Sudan.^5^

MenAfriVac®, a low-cost, temperature-stable meningococcal A conjugate vaccine (MACV), has been used for mass vaccination campaigns in the meningitis belt since 2010. MenAfriVac® is delivered in a single dose, with antibodies persisting significantly above baseline for at least 5 years.^6^ Rapid rollout began in the highest-risk areas of Burkina Faso, Mali, and Niger and followed throughout the region. Epidemiologic risk profiling tools were used to plan the sequence of country introductions and determine whether the vaccine should be introduced nationwide or targeted to high-risk areas within each country.^5^^,^^7^ By the end of 2019, almost 350 million residents of the African meningitis belt age 1–29 years had received a dose of MACV through a series of highly successful, well-received campaigns.^8^ A surveillance study across nine countries in the meningitis belt from 2005 to 2015 found that the incidence of suspected meningitis declined by 57% in vaccinated versus unvaccinated populations, and confirmed MenA infection decreased by over 99%.^9^

Immunising 1- to 29-year-olds at once led to virtual elimination of MenA carriage and interrupted transmission of the bacteria.^10^ The success of the MenA program hinges on continued herd immunity. WHO recommends that countries that have completed mass campaigns introduce MenA conjugate vaccine into their routine immunisation (RI) programs within 1–5 years, along with a catch-up campaign for birth cohorts born after initial vaccination but prior to the implementation for RI.^11^ In their global roadmap for “defeating meningitis by 2030”, WHO recommends that this RI introduction occur in all meningitis belt countries by the end of 2023.^12^ As of the end of 2021, twenty-four of the twenty-six countries in the region have conducted mass immunisation campaigns, and twelve of them have completed introduction of MACV into their national RI schedules.^13^ Delayed introduction of RI and catch-up campaigns is predicted to result in loss of herd protection and lead to risk of resurgence of epidemics, although a large resurgence would not be expected until around 15–20 years after the initial campaign.^14^ Modelling studies have suggested that sustained RI at a coverage greater than 60% is the best way to protect against MenA epidemics.^14^

Post-campaign coverage data in select meningitis belt countries have been previously published by Bwaka et al.^5^ However, no study currently exists estimating total coverage of MACV, including campaigns plus RI, across all countries where it is administered. This study builds on Bwaka et al.'s work, synthesizing campaign coverage information and RI estimates and produce estimates of MACV coverage in all countries where it is administered routinely or as part of a campaign, from 2010 and extends through to 2021.

## Methods

### Study design

**Fig. 1:**
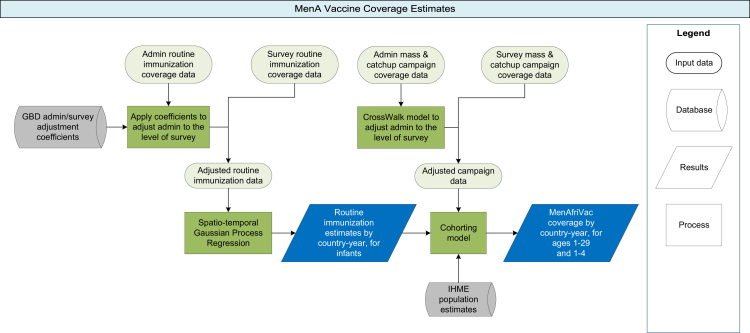
Diagram of methods. Green squares indicate a modelling step, blue parallelograms indicate results, light green ovals indicate data, and grey cylinders indicate GBD (Global Burden of Disease Study) results used as input to this model.

A flowchart of all methods is provided in Fig. 1. Routine immunisation coverage was modelled for twelve meningitis belt countries. These countries are: Burkina Faso, Central African Republic, Chad, Côte d’Ivoire, Eritrea, the Gambia, Ghana, Guinea, Mali, Niger, Nigeria, and Sudan. Total coverage of campaign data plus routine immunisation was modelled for the twenty-four of the twenty-six meningitis belt countries which have introduced any MenA immunisation as of 2021. These countries are those listed for RI modelling, plus: Benin, Burundi, Cameroon, Democratic Republic of the Congo, Ethiopia, Guinea Bissau, Kenya, Mauritania, Senegal, South Sudan, Togo and Uganda.^15^ Rwanda and Tanzania are in the meningitis belt but have not introduced any immunisation activity, so are not included in modelling.

Data were not obtained from subjects for the Global Burden of Diseases, Injuries, and Risk Factors Study. Instead, we used pre-existing, publicly available, de-identified datasets that include but are not limited to administrative and survey-based vaccine coverage reports. Data are identified through online searches, through outreach to institutions that hold relevant data such as ministries of health, or through individual collaborator reference and identification. Most data used are publicly available. Therefore, informed consent is not required. This study was approved by University of Washington's Human Subjects Division Study ID: STUDY00009060.

Our study follows the Guidelines for Accurate and Transparent Health Estimates Reporting (GATHER; Supplementary Table S3). The findings of this study are supported by data available in public online repositories and data publicly available upon request of the data provider. Details of data sources and availability are publicly available in the Global Health Data Exchange (https://ghdx.healthdata.org/record/ihme-data/sub-saharan-africa-menafrivac-estimates-2010-2021). The full output of the analyses can be found in Supplementary Table S4 and at the abovementioned website.

### Data sources

**Fig. 2:**
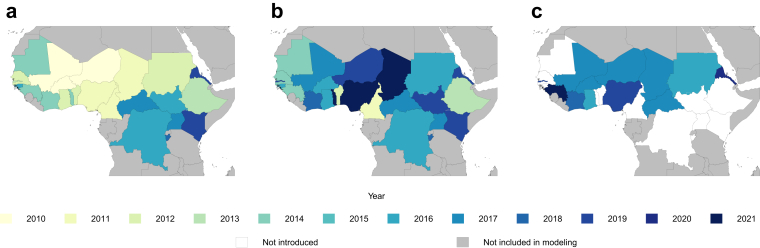
Meningococcal A containing conjugate vaccine (MACV) in the meningitis belt, by year of introduction. (a) Shows the year of the first mass campaign. (b) Shows the year of the most recent campaign (mass or catchup). (c) Shows the year of introduction into routine immunization schedules.

For the two main types of MACV delivery, campaigns and RI, data were gathered separately. Data on campaigns, both initial and catch-up, were obtained from country reports summarised in Bwaka et al., WHO Weekly Epidemiological Report publications, and, for year 2021, unpublished WHO data on the size of the target population for the campaign in Nigeria (Bita, unpublished).^5^^,^^13^^,^^16^^,^^17^ For routine immunisation, WHO data on national vaccine schedules, as well as location-specific introduction years of MACV into the schedule, were used in determining which location-years to model (Fig. 2).^18^^,^^19^ We utilised administrative data on RI coverage reported by countries annually to the WHO Immunization Data portal through the WHO/UNICEF Joint Reporting Form on Immunization (JRF), as well as survey data reported from sources in our systematic review.^5^^,^^20^, ^21^, ^22^, ^23^ Administrative data was used preferentially over the official estimates set out by countries due to higher completeness of administrative data in meningitis belt countries, and inconsistency in the methods of calculation of official coverage between countries, although the two sets of data were similar (Supplementary Fig. S3). In addition, a systematic literature review was conducted using the Institute for Health Metrics and Evaluation's (IHME) Global Health Data Exchange (GHDx) and PubMed to find additional sources on routine immunisation coverage, with search string and complete inclusion and exclusion criteria described in the Supplement.

Administrative vaccination coverage estimates are calculated by dividing the number of doses administered (via RI or campaign) by the estimated number of people in the target population for vaccination.^24^ These estimates may be biased and tend to overestimate coverage, due to mismatches between numerator and denominator or inaccurate/incomplete estimates of population or services delivered.^25^ Survey methodology varies, but post-campaign surveys were typically conducted by independent monitors at least 1 month after the end of the campaign, using either the WHO EPI stratified cluster survey method or the lot quality assurance sampling method, with additional information provided in the Supplement.^26^ For both campaign and RI data, survey data was utilised preferentially over administrative data; when both survey data and administrative data were available for the same country-year, the survey point was used as input data and the administrative point was discarded. When survey data was unavailable, administrative data was adjusted to the level of the survey data using the bias adjustment described below. An exception to the preferential handling of survey data occurred when the implied number of survey doses calculated using IHME population estimates exceeded the number of admin doses reported. In this case, the survey data was deemed implausible and admin data was used.

Because the vast majority of data were available only for both sexes together, we modelled coverage among both sexes. When only sex-specific data were available, male and female coverage values were averaged. Population estimates, utilised in the cohort model, were from the IHME Global Burden of Disease Study.^27^ In total, we utilised 49 country-years of routine immunisation data and 53 country-years of campaign data.

### Administrative bias adjustment: Campaign data

To account for systematic bias in administrative data, we quantified the degree of bias for each administrative data point and adjusted it prior to modelling with the CrossWalk package in R.^28^ Using survey data as the gold standard, we created matched pairs of administrative (alternative) and survey (reference) observations from Bwaka et al. The matching criteria were that each pair of points should reflect the same location-year-campaign. We then took the difference in logit space and used this as the dependent variable in a statistical model. The dependent variable represents the logit-scale degree of bias in administrative data. The model included log-transformed population size as a covariate because increasing size of the target population is positively associated with a larger discrepancy between administrative and survey data in this dataset. This aids in out-of-sample prediction for locations without matched pairs. To obtain the estimated unbiased value for an administrative data point, we applied the predicted adjustment factor by subtracting it from the original observation in logit space. Any adjusted administrative data point still exceeding 100% was coerced to 99%. Details of this adjustment can be found in the Supplement.

### Administrative bias adjustment: RI data

When estimating vaccination coverage, survey data, such as USAID's Demographic and Health Survey (DHS) and UNICEF's Multiple Indicator Cluster Survey (MICS), are often considered more reliable than administrative data.^29^ However, except for two recent DHS surveys in Mali 2018 and the Gambia 2019–20, DHS and MICS do not ask about MACV, making survey data for MACV coverage rare. For RI data, we again implemented bias adjustments for administrative data using survey data as the gold standard. However, due to the lack of RI survey observations, we used bias adjustment coefficients for other vaccines estimated as part of the Global Burden of Disease Study.^30^ These coefficients represent a ratio of administrative:survey, modelled as a country- and vaccine-specific, time-varying estimate of the relationship between administrative and survey coverage estimates. Coefficients for Bacillus Calmette−Guérin (BCG), third dose diphtheria tetanus pertussis (DTP3), first dose measles-containing vaccine (MCV1) and third dose polio (Pol3) were averaged to approximate the adjustment coefficient for MACV (Supplementary Figs. S1 and S2). The RI administrative data adjustment was performed by multiplying this adjustment coefficient times the raw RI administrative data to get adjusted RI administrative data.

### Routine immunisation coverage estimation

Using all available data on RI, including survey data and bias-corrected administrative data, MACV coverage was modelled using Spatiotemporal Gaussian Process Regression (ST-GPR), a multistage mixed effects model that starts with a linear regression and adds smoothing over space and time. The technical details of ST-GPR can be found in the Appendix (Supplementary Appendix 1, pages 34–38) of a previous publication.^31^ In short, this process accounts for the variance of each data point and incorporates this into the estimation of uncertainty in the ST-GPR result. Additionally, it uses covariates and data from neighboring locations to generate estimates for all location-years even where data is not available. The covariates used were the Global Burden of Disease Study's healthcare access and quality (HAQ) index and estimates of mortality rate from conflict and terrorism.^1^ To estimate 95% uncertainty intervals (UIs) for vaccine coverage estimates, 1000 random draws were sampled from the modelled posterior distribution and the ordinal 2.5th and 97.5th percentile of draws were used as the upper and lower bounds of the interval for each measure.

Only single dose MACV coverage was modelled, whether it was mass campaign or RI. RI coverage was coerced to zero in country-years where MACV was not present in the schedule. The RI model is age-agnostic: the data and results reflect coverage at the target age of RI, which varies from 9 to 18 months across countries.

### Overall vaccination coverage estimation: Routine plus campaigns

Within the meningitis belt, MACV has been delivered through initial mass campaigns, catch-up campaigns, and RI. Initial mass campaigns targeted children and adults between 1 and 29 years, RI targets infants between 9 and 18 months, and catch-up campaigns target children of varying ages depending on the campaign, but typically under 10 years.^5^ We used a cohort model to account for vaccine coverage by age due to campaigns and RI together.^32^ This cohort model operated at the single-year-age group level for all ages 1–29, creating simulated age cohorts and following them over time, from 2010 through 2021. The 1–29 age group was selected because it was the initial target age for MACV campaigns and because meningococcal carriage in the meningitis belt peaks between ages 5 and 19.^33^ Additionally, results are presented for aggregations in the 1–4 age group because children under 5 experience the highest rates of meningococcal meningitis incidence and mortality.^1^

The cohort model had four components. First, for mass campaigns, it applied the coverage from the campaign to all cohorts in the 1–29 population. Second, it aged the population up each year, adding new non-immunised 1-year-olds to the group and removing 30-year-olds. Third, it incorporated catch-up campaigns. Fourth, it incorporated the estimates of infant RI that were previously discussed, calculating the proportion of 1- and 2-year-olds who are vaccinated.

Each model started in the year 2010 and assumed zero coverage in a country until the first year with immunisation activity. In the year of the first mass campaign, all age cohorts falling into the target age group were assigned a coverage value reflecting coverage from that campaign. If post-campaign survey data was available for that location-year, the coverage value was taken to be the survey value from survey data. If only administrative data was available, the coverage value was taken to be the value from administrative data, adjusted for bias as previously described. In the case of multi-year campaigns with one data point for coverage at the end of the campaign, coverage from the campaign was modelled to increase linearly over the years of the campaign.

For each year following, the population aged up 1 year and new children turning one in a given year entered the group. Population was recalibrated to IHME estimates for each year to account for mortality and migration. If it was any year prior to RI introduction, new infants entered with an immunisation coverage of zero. If it was a year in which RI was established, ST-GPR was used to calculate coverage for 1- and 2-year-olds. To account for the varying ages of introduction of RI, year-end coverage for each age cohort from RI activity was calculated as the sum of the portion of each cohort who received RI in the current year and the portion of the cohort who received RI in the previous year:

If the immunisation target age is <12 months:

$${Coverage}_{C1,y}=\left({RI}_{T,y}{*frac}_{C1,T,y}\right)+\left({RI}_{T,y-1}*\left(1-{frac}_{C1,T,y}\right)\right)$$

If the immunisation target age is 12–24 months:

$${Coverage}_{C1,y}=\left({RI}_{T,Y}{*frac}_{C1,T,y}\right)$$

$${Coverage}_{C2,y}=\left({RI}_{T,y}{*frac}_{C2,T,y}\right)+\left({RI}_{T,y-1}*\left(1-{frac}_{C2,T,y}\right)\right)$$

where:

$$frac_{C,T,y}=T-floor\left(T\right)$$

where y is the year of interest, C is the age cohort of interest (C1 for 1-year-olds, or C2 for 2-year-olds), T is the immunisation target age in years, RI_T,y_ is modelled RI at the target age T estimated with ST-GPR in year y, and frac_C,T,y_ is the fraction of infants in age cohort C who reach the target age for immunisation T in year y.

When a mass preventive or catch-up campaign was conducted, we assumed that all age cohorts falling into the target age group of that campaign were equally likely to receive a dose and assigned them a coverage value reflecting the coverage from that campaign. In the case where a cohort targeted by a catch-up campaign and an age group already immunised from a previous mass campaign or RI overlapped, an untargeted, unbiased strategy was used to determine the likelihood that a vaccine dose will go to a not previously immunised child. This assumes that a child's previous vaccination status does not influence his or her likelihood to receive a vaccine in the campaign.^32^

The result is a time series of immunisation coverage that incorporates mass campaign data, catch-up campaign data, and RI estimates. Single year age group coverage was combined across years to get overall coverage for the 1–29 and 1–4 age groups, calculated for each country-year, from 2010 to 2021. To estimate uncertainty, these calculations were performed at the 1000-draw level. We used estimates of uncertainty around campaign data (method described in Supplement) to generate 1000 draws of these data in a binomial distribution. We also used 1000 draws from ST-GPR results, which had incorporated the uncertainty of the routine immunisation data. This propagated uncertainty from both campaign and routine immunisation data into the result.

### Model notes and assumptions

In several countries, MACV campaigns targeted only a subset of the population living in the highest-risk areas of the meningitis belt. Therefore, we produced two versions of this model: one reporting coverage in the whole country, and another reporting coverage in high-risk areas. The high-risk area analysis is presented below, and the full-country analysis is available in the Supplement. For the purpose of this analysis, we defined high-risk areas as those that were targeted in the initial campaigns in Bwaka et al.^5^ In countries where the initial campaign targeted the entire population, results from the high-risk area analysis and full-country analysis are identical. To derive whole country coverage when only coverage in a subset of the population was reported, such as the high-risk coverage, we adjusted the value downward to reflect the portion of the population living in those regions; these adjustment values are reported in the Supplement (Supplementary Table S1). Immunisations during a campaign were assumed to be distributed equally to all age cohorts within the target age of the campaign. For the high-risk denominator model, RI coverage levels were assumed to be equal in high-risk areas as in the total population, as RI rollout was nationwide for modelled location-years. Coverage values for a given year reflect estimates as of the last day of that year.

### Role of the funding source

The funder of the study had no role in study design, data collection, data analysis, data interpretation, or writing of the report.

## Results

### Routine immunisation trends and coverage

**Fig. 3:**
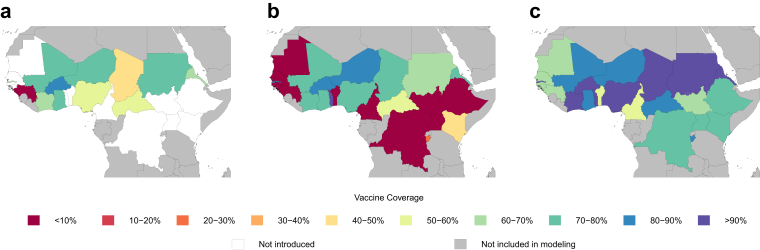
Coverage estimates for high-risk country populations for the meningitis belt, at year end 2021. (a) Shows the routine immunisation values for the target age, which varies by country from 9 to 18 months. (b) Shows combined coverage estimates for mass campaigns, catch-up campaigns, and routine immunisation coverage combined for ages 1–4. (c) Shows analogous combined coverage estimates for ages 1–29.

We estimated that eight of twelve meningitis belt countries with completed RI rollout have coverage above 60% in 2021, with Burkina Faso having the highest estimated coverage, at 87.3% (95% UI 85.0–89.3). Côte d'Ivoire, Eritrea, the Gambia, Ghana, Mali, Niger, and Sudan also surpassed the sixty percent threshold. The Central African Republic, Chad, and Nigeria had mean estimated coverage values between forty and sixty percent, and Guinea had coverage below forty percent (Fig. 3a). As of the end of 2021, two meningitis belt countries, Togo and Benin, were beginning but had not completed their RI rollout, and twelve countries had not yet introduced RI.

**Fig. 4:**
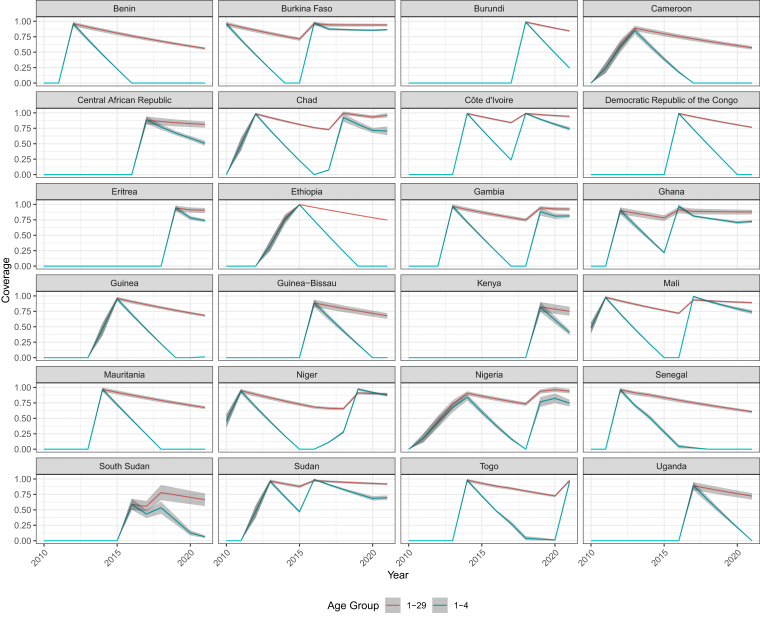
Coverage estimates for high-risk country populations for the meningitis belt, for 1–29 and 1–4 age groups, from 2010 to 2021. Year−end coverage values shown for each year.

### Age cohort coverage in the meningitis belt

As of 2021, twenty-four of the twenty-six countries in the meningitis belt had initiated MACV mass vaccination campaigns, RI, or both. Although timing of vaccination campaigns by country was determined by risk profiling tools, levels of peak vaccination coverage did not correlate with pre-introduction disease burden (Supplementary Fig. S4). Among children aged 1–4 in 2021, the three countries with the highest estimated coverage in high-risk areas were Togo, Niger, and Burkina Faso, with 96.0% (95% UI 92.0–99.0), 87.2% (95% UI 85.3–89.0), and 86.4% (95% UI 85.1–87.6), respectively (Fig. 4). These had high coverage values driven by an initial successful mass immunisation campaign, followed by a catch-up campaign, followed by introduction of RI into the infant schedule, in 2017 for Niger and Burkina Faso and 2021 for Togo, though Togo's introduction in late December 2021 is not captured in this model. Eight countries – Benin, Cameroon, Democratic Republic of the Congo, Ethiopia, Guinea-Bissau, Mauritania, Senegal, and Uganda – are estimated to currently have zero coverage for children 1–4 (Fig. 3b) because catch-up campaigns or RI have not yet followed the initial mass vaccination push.

Even in countries with no coverage in young children, coverage from previous campaigns in older children and adults can provide lasting protection via herd immunity. Across the meningitis belt, countries with the highest estimated coverage for ages 1–29 in high-risk areas are Togo, Chad, and Côte d’Ivoire, with 97.6% (95% UI 95.4–99.4), 96.0% (95% UI 92.5–99.6), and 94.3% (95% UI 92.6–95.4) coverage, respectively (Fig. 4). Coverage proportions were higher in this 1–29 age group compared to the 1–4 age group, with a median coverage of 82.9%. In twenty-two countries, immunisation coverage surpassed 60%, with no country showing zero coverage. In comparison, the 1–4 group had a median coverage of 45.6%, with eleven countries surpassing 60% coverage (Fig. 3b and c).

For the analysis including the full country populations in the denominator, not only those living in high-risk areas, select countries that targeted only a subset of their population for MACV campaigns have much lower nationwide coverage estimates. For example, coverage of the 1–4 age group using the high-risk population of Benin following a mass campaign in 2012 was estimated to be 96.0% (95% UI 92.0–99.0). However, nationwide coverage of the full population in that year was 35.5% (95% UI 26.0–45.0) (Supplementary Fig. S6). Niger and Burkina Faso have the highest full population coverage estimates in the 1–4 age group; these estimated values are the same as in the high-risk analysis because their campaigns were nationwide. Togo, which had the highest coverage in the high-risk population for the 1–4 age group, only targeted a portion of its population for campaigns and ranks 8th in the full population estimates, with an estimated coverage of 69.9% (95% UI 61.0–79.0) in 2021 (Supplementary Figs S5 and S6).

The high degree of protection in all ages in countries such as Burkina Faso, Mali, and Sudan comes from mass campaigns followed by early catch-up campaigns, and finally completed with high-coverage RI introduction as of 2021. Other countries, which have had past mass campaigns but have not followed up with catch-up or RI, have low immunisation coverage in children. In these locations, such as Ethiopia, many young adults remain protected from earlier campaigns, with a coverage value of 74.6% (95% UI 74.1–74.8) in the 1–29 age group. In comparison, because there has been no immunisation activity in the past 4 years, the 1–4 age group in Ethiopia has a coverage value of zero (Fig. 4).

### Time trends in coverage in the meningitis belt

In many countries, coverage quickly jumped from zero to a high level following a successful initial mass immunisation campaign in an unvaccinated population. This coverage dropped off over time when introduction into the immunisation schedule did not immediately follow the campaign. This drop off was steeper in the 1–4 cohort than the 1–29, as a larger proportion of the 1–4 cohort aged out every year and was replaced by new susceptible children in the absence of an effective RI program. In some countries, that decline was sustained, while in others, the decline was offset partially or completely by catch-up campaigns or RI. In Ghana, for example, campaigns targeted only a subset of the population. RI was initiated early, in 2017, with an estimated 77.0% (95% UI 73.4–80.3) coverage in 2021, above the 60% suggested threshold for epidemic prevention. By 2021, overall coverage began to trend upward because of the influence of new RI, with coverage at 72.4% (95% UI 70.0–74.5) for ages 1–4 (Fig. 4).

## Discussion

In this study, we extended Bwaka et al.'s previous work in reporting MACV campaign status, synthesizing these campaign coverage data with RI data to generate comprehensive coverage estimates. As such, this study provides an improved understanding of how MACV coverage varies by age from 2010 to 2021 in twenty-four meningitis belt countries that have administered it through RI, campaigns, or both.

We found that the three countries with the highest coverage among 1- to 4-year-olds, Togo, Burkina Faso, and Niger, are those that have followed WHO recommendations: first conducting a mass campaign, followed by RI plus a catch-up campaign. However, even countries that have implemented this strategy, including Mali and Chad, have seen their immunisation coverage levels fall from their post-campaign peak over time. RI coverage in these locations is much lower than coverage from campaigns. Twenty-eight of thirty campaigns with administrative data reported in Bwaka et al. report coverage values over ninety percent, compared with zero out of eleven administrative reports of RI. Only four RI reports show coverage over eighty percent.^5^

While one-time campaigns were highly successful in delivering immunisations, they cannot stand as the permanent, sole strategy. These drops in overall coverage in countries that have transitioned to routine MACV immunisation indicate a need to strengthen RI delivery systems. In Ghana, a survey of health care professionals administering vaccines found that coverage for vaccines given in the second year of life, including MACV, is generally lower than for those given in the first year.^34^ In Burkina Faso, where MACV is also administered in the second year of life, caregivers most commonly reported lack of awareness about the vaccination visit as the reason for nonvaccination.^21^ These two studies suggest that reaching and maintaining high levels of MACV coverage may require additional resources in countries that schedule the vaccine in the second year of life.

Our estimates underscore the tremendous public health success of MACV.^9^ Across the meningitis belt, in fully vaccinated populations, the incidence of MenA declined by 99% from one year before vaccination to three years after vaccination.^9^ Furthermore, MenA has been responsible for zero reported outbreaks in countries that introduced MACV since 2011.^35^ However, sporadic MenA cases have still been reported in unvaccinated children, emphasising the need for continued RI against the pathogen.^5^^,^^36^ This study captured RI in twelve countries that have completed introduction as of the end of 2021; additionally, Benin and Togo plan to complete RI introduction in 2022.^13^ These estimates help highlight where gaps in immunisation remain and emphasize the need for rapid introduction and sustainable implementation of RI into at-risk countries where early campaigns are currently the only source of population coverage.

The COVID pandemic, and its associated strain on healthcare systems, have disrupted RI in 2020 and 2021 in most countries.^37^ Fortunately, disruptions to MACV RI were minimal: of nine meningitis belt countries that reported RI data to WHO for 2019 through 2021, six report higher coverage values in 2021 than in 2019.^20^ Even if a 1-year disruption in routine MACV immunisation were to occur, it would be unlikely to have a substantial detrimental effect on meningococcal infection even in countries with high epidemic potential, due to the persistence of coverage from earlier campaigns in the 1- to 29-year-old populations.^38^

Beyond serotype A, non-A meningococcal epidemics are on the rise in the meningitis belt.^9^^,^^39^ Between 2009 and 2017, MenC and MenW replaced MenA as the most frequently isolated meningitis pathogens in the region. MenX, while comparatively rare, has increased proportionally in recent years, from 2.7% of confirmed cases in 2013–2016 to 22.0% of confirmed cases in 2017.^40^ These outbreaks emphasise the need for the development and introduction of a multivalent meningococcal vaccine for use in Africa. One such vaccine candidate against MenACYWX was found to be effective in a recent trial in infants in Mali.^41^ This would be the first approved vaccine against meningococcal serotype X, for which no vaccine currently exists. Gavi approved expansion of the existing meningococcal program in Africa to support serogroup ACW-containing meningococcal conjugate vaccines in 2018, but implementation has not yet been rolled out.^42^ In the long term, high routine coverage with multivalent conjugate vaccines would be an optimal approach to meningococcal meningitis control.^12^ In the near term, modelling studies suggest that the risk of epidemic MenA resurgence may be low in the first 15 years following mass vaccination initiation.^14^ Moreover, the substantial population-level immunity conferred by MenA vaccination activities to date and the availability of MenA vaccines may allow countries to respond strategically with targeted vaccination activities if new outbreaks should occur. Countries must therefore balance the timing of new vaccine availability against the build-up of susceptible individuals — and account for competing logistical priorities — when deciding whether to prioritise introduction and scale-up of MenA or await the availability of a multivalent conjugate vaccine such as ACYWX. Efforts to introduce and scale-up meningococcal conjugate vaccines must also be integrated with broader efforts to strengthen RI systems and ensure equitable access to vaccines for all.^12^

The study has several limitations. Coverage was estimated at the national and high-risk area level only. Further data would be needed to create estimates at the subnational or district level, such as that which has been done for measles.^43^ While the RI estimation stage of the model incorporated both administrative and survey data, the cohort stage incorporated only one data point per location-year, utilising survey data if available, and adjusted administrative data if not. Although survey data is the gold standard, recall bias may skew estimates upward or downward. In addition, selection bias can inflate survey estimates, which are unlikely to accurately capture refugee or internally displaced populations. Since these populations are also at higher risk for infectious diseases such as meningitis, additional work is needed to quantify vaccine delivery in these areas. Due to a lack of survey data on MenA RI coverage, we pooled estimates of similar bias in BCG, Pol3, MCV1, and DTP3 to adjust for bias in administrative data on MenA RI coverage. This approach assumes that the drivers in bias for MenA – including numerator and denominator error – are similar to those for other routine childhood vaccines. Future estimates would be strengthened by directly estimating bias in MenA RI administrative data, if additional survey data become available.

In addition, due to a lack of age-sex-specific campaign data, we assumed equal coverage for both sexes and all ages within the campaign's target age range. Age-specific data on coverage was only available from three sources representing two countries: post MACV campaign surveys in Burkina Faso and Niger have shown that adolescents over the age of 15 have lower vaccine uptake than younger children.^44^, ^45^, ^46^ Given the limited availability of age-specific data, we did not apply these age patterns to other campaigns, but rather assumed that coverage was the same for all age groups in the eligible population for a campaign. For sex-specific data, one survey showed a larger disparity in vaccine coverage by age in males than females, suggesting a possible role of male sex as an effect modifier, and a decrease in young adult male attendance at vaccine clinics has been qualitatively observed.^26^^,^^44^ In other surveys, there was no significant association in vaccine uptake between sexes.^45^, ^46^, ^47^ More precise information about age- and sex-specific vaccine uptake during campaigns would substantially improve the accuracy of future models. Similarly, this analysis assumes no correlation between prior vaccination status and receipt of a vaccine in a campaign. If future campaign surveys were to include information on prior vaccination status, this would allow for better assessment of population-level vaccination coverage following campaigns and strengthen modelling efforts. Finally, this model quantifies coverage, not immunity, and does not account for waning immunity over time.^48^

Combining RI and campaign data, we summarised overall MenA vaccine coverage in twenty-four meningitis belt countries. Our results, which are comprehensive over location and time, highlight the success of early MenA campaigns while also showing the need for sustained immunisation activity into the present. In August 2021, the WHO regional committee for Africa endorsed a framework for the implementation of the global strategy to defeat meningitis by 2030 in the African region. The framework highlights a strong commitment from the region to sustain the virtual elimination of MenA epidemics.^49^ When used in conjunction with meningitis microbial surveillance and estimation, these strategies can help identify regions for prioritisation of routine immunisation or campaigns to meet WHO's goal of defeating meningitis by 2030.

## Contributors

R.G.B., J.F.M., & J.S. conceptualized the project. R.G.B., A.A.B.F., A.M.B., N.C.G., & J.S. participated in data curation, and R.G.B., N.C.G., and J.S. conducted the formal analysis and investigation. A.A., R.G.B., N.C.G., J.F.M., E.L.B.R., A.N.S., R.J.D.S., & P.Z. developed the methodology. J.F.M. & R.J.D.S. contributed to software development. H.H.K., S.I.H., & J.F.M. supervised the project. A.M.B. & A.L. provided validation of the results. R.G.B. created visualizations. R.G.B. & E.L.B.R. wrote the original draft, and A.M.B., A.A.B.F., E.H., S.I.H., H.H.K., A.L., J.F.M., J.M.M., E.L.B.R., A.N.S., R.J.D.S., A.V., & C.W. provided critical review, commentary and revision during the writing process. R.G.B., H.H.K., & J.F.M. had full access to and verified the underlying data used to generate the estimates presented in this article. All other authors had access to and reviewed the estimates as part of the research evaluation process, which included additional formal stages of review. The corresponding author had final responsibility for the decision to submit for publication.

## Data sharing statement

Metadata for all sources of data analysed in the current study can be freely accessed in the Global Health Data Exchange (http://ghdx.healthdata.org/) upon publication.

## Declaration of interests

IHME is primarily funded by the Bill and Melinda Gates Foundation. JM & AS additionally report receiving funding from Gavi. CW reports that the Meningitis Research Foundation has received grants in support of their charitable objectives from GSK, Pfizer, Sanofi Pasteur, Serum Institute, and Tableau foundation. CW reports that the Meningitis Research foundation has received consulting fees from GSK within the last 4 years.

## Appendix A Supplementary data

Supplemental Figures S1–S6 and Tables S1–S4

Caption for Supplementary Material
