# Supplementary material for: Meningococcal A conjugate vaccine coverage in the meningitis belt of Africa from 2010 to 2021: a modelling study
Source: eClinicalMedicine. 2023 Jan 5;56:101797. doi: 10.1016/j.eclinm.2022.101797 (PMC9985031; doi:10.1016/j.eclinm.2022.101797)
Supplement: Supplemental Figures S1–S6 and Tables S1–S4 [file mmc1.pdf]

Supplementary Material

Contents

Data 2
Routine immunisation 2
PRISMA diagram for routine immunization data 3
Campaigns 3
Methods 4
Estimating the proportion of the population considered to be high-risk 4
Estimating the full-population post-campaign coverage when coverage in a sub-population is reported 4
Estimating variance for data points 4
Bias adjustment for campaign data 5
Bias adjustment for routine immunisation data 6
Adherence to reporting guidelines 8
Results 10
Supplemental Figures 10
Supplemental Table 13

Data

Routine immunisation

PubMed was searched for available literature data pertaining to MACV coverage on July 6th, 2020, using the search string:

((("meningococcal" OR "meningococcal infections" [Mesh] OR "meningococcal infections" OR neisseria meningitidis [Mesh] OR "neisseria meningitidis" OR "meningitis, meningococcal" [Mesh] OR "meningococcal meningitis") AND ("immunization" [Mesh] OR "vaccines" [Mesh] OR "vaccination" [Mesh] OR "immunization" OR "immunizations" OR "immunisation" OR "immunisations" OR "vaccine" OR "vaccines" OR "vaccination" OR "mass vaccination" OR "immunization programs" [Mesh] OR "immunization programs")) OR ("menafrivac" OR "meningococcal vaccines" OR "meningococcal vaccines" [Mesh])) AND ("vaccination coverage" [mesh] OR "prevalence" [mesh] OR "coverage" OR "uptake" OR "prevalence" OR "immunization rate" OR "vaccination rate" OR "immunisation rate")

This search returned 1028 results. In addition, the Institute for Health Metrics and Evaluation's Global Health Data Exchange, or GHDx (http://ghdx.healthdata.org/), was searched on July 13th, 2020 for the keyword "meningococcal vaccines". The GHDx yielded 252 results.

We included any study in the routine immunisation modelling step that met at least one of the following criteria:

1. Includes data from one of the twenty six countries defined as a meningitis belt country by the WHO[1]
2. Includes data from a country that includes meningococcal A conjugate vaccines (MenA, MenAC, MenA_conj, MenACW, MenACWY-135 conjugate) in its national immunisation schedule for all people in the years covered by the study
3. Reports population-level data on routine meningococcal conjugate A immunisation coverage
4. Uses a probability-based sampling methodology
5. Includes coverage data for the entire year of interest (as compared to a quarterly report, for example)

We excluded any study that fell into one of the following categories:

1. Pre-2005 (when the first meningococcal conjugate vaccine was approved)
2. Only includes data on vaccines administered in the private sector
3. Only includes data on mass campaigns or catch-up campaigns*
4. Only includes data on reactive campaigns, or campaigns occurring in a small area in response to an outbreak using vaccines from the emergency stockpile
5. Not nationally representative
6. Only assess coverage within a subgroup not representative of the general population (ie immunocompromised patients, asplenic patients, Hajj travelers, prisoners)
7. Sample size of fewer than 50 people
8. Includes aggregated data from more than one country and does not report that data separately by country
9. Examines a meningococcal vaccine that does not contain MenA, such as MenB or MenC only vaccines
10. Only measures polysaccharide vaccine coverage
11. Reports vaccine coverage in a country prior to the year of MACV introduction in that country
12. Reports vaccine coverage in a country where MACV is not part of the routine schedule
13. Reports second dose or booster dose coverage only

*Excluded in routine immunisation modelling step only.

Of 1280 total results, six were selected for inclusion in routine immunisation modelling. This encompassed 49 country-years of routine immunisation data, given that several sources, most notably the WHO immunisation data portal, included multiple country-years of data. Our routine immunisation data was 98% complete, as there is a total of 50 country-years of potential data, given the years of MACV introduction by country; the only missing country-year was the Gambia in 2020.

PRISMA diagram for routine immunization data

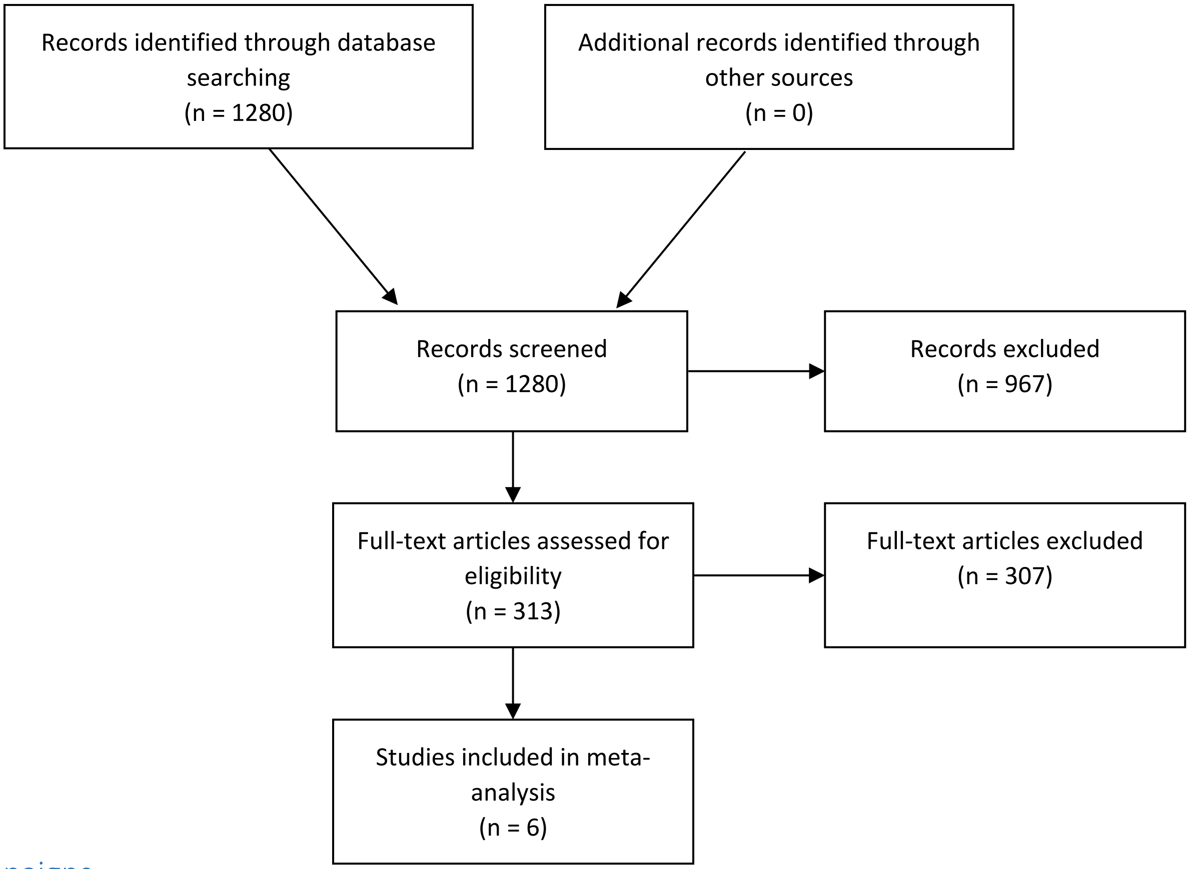

Campaigns

Data on mass campaigns, both initial and catch-up, were extracted primarily from Bwaka *et al.* Table 2.[2] In a few instances, the years listed in Table 1 and Table 2 were not consistent. In these cases, original WHO Weekly Epidemiological Reports were consulted. In addition, coverage values too recent to be reported in Bwaka et al. were extracted from original WHO reports.[1,3] When only two of the three values of target population, number vaccinated, and administrative coverage were provided, the third was calculated. In total, 41 administrative data points were available, comprising all known MenAfriVac campaigns. All these data were reported to have 100% completeness of administrative data reporting. These points covered 53 country-years, as some campaigns spanned multiple years. 22 out of 41 campaigns had survey data available. For those surveys, three had information available on response rate: 95.1% in Cameroon in 2013, 99.1% in Togo in 2014, and 89.0% in Uganda in 2017 (Bwaka, unpublished).

The post-campaign coverage surveys (PCCS) were carried out independently by either national institutions such as public health/academic institutions, national bureau of statistics or institutes or consulting firms, with the support of a WHO-international hired consultant. For the surveys, the 2015 WHO Vaccination Coverage Cluster Survey Reference Manual and the 2018 revised WHO Vaccination Coverage Cluster Survey Reference Manual were the reference documents, and the methodology specified in the guide was the one used for all the surveys.[4] This guide also describes the implications and considerations for sample size, resources and quality control of stratifying surveys to different levels of the health system.[5] Survey supervisors were generally recruited from line ministries and independent institutions, and cascaded trainings were carried out from central level to the district level to ensure quality supervision of data collection and implementation of the methodology. Field surveyors were recruited among either students from medical institutions, personnel from line ministries or independent Non-Governmental Organizations (NGOs), Civil Society Organizations or staff of Ministry of Health but not involved in immunisation programs.

Methods

Estimating the proportion of the population considered to be high-risk

For this analysis, the definition of high-risk was determined by the places that were targeted in the initial MenAfriVac campaign, i.e., we assumed that the targeted area comprises the whole high-risk population. The target population in these areas were extracted from Bwaka *et al.* Table 2.[2] We used IHME estimates for ages 1-29 from the campaign year as the total country population. A subnational map of the specific locations targeted by MenAfriVac campaign rollout through 2015 has been previously published elsewhere.[6] Once these proportion scalars were calculated, they were assumed to be constant over time. They are listed below:

Country	High risk area	Age	Target population	Initial campaign year	IHME population	High-risk Proportion
Benin	5 northern Regions	1-29 yrs	2,595,654	2012	6,993,309	37%
Côte d'Ivoire	25 northern districts	1-29 yrs	4,271,669	2014	15,645,489	27%
Cameroon	4 northern regions	1-29 yrs	6,727,388	2011-2013	16,830,105	40%
DRC	6 eastern provinces	1-29 yrs	18,205,784	2016	54,108,488	34%
Ghana	3 northern Regions	1-29 yrs	3,098,348	2012	17,232,997	18%
Guinea	17 northern & eastern districts	1-29 yrs	3,005,423	2014-2015	7,802,219	39%
Kenya	5 districts: Mandera, Marsabit, Turkana, Wajir and West Pokot	1-29 yrs	3,122,735	2019	32,417,267	10%
Mauritania	33 southern districts	1-29 yrs	1,610,523	2014	2,472,553	65%
Nigeria	17 northern States	1-29 yrs	83,695,197	2011-2014	128,547,396	65%
Senegal	8 northern regions	1-29 yrs	4,383,255	2012	8,815,513	50%
Togo	42 northern districts	1-29 yrs	2,754,189	2014	4,652,412	59%
Uganda	39 northern districts	1-29 yrs	6,899,267	2017	28,239,239	24%

Table S1: Estimated proportion of national population living in high-risk regions, by country.

Estimating the full-population post-campaign coverage when coverage in a sub-population is reported

In some cases, survey or administrative post-campaign coverage results were reported for a portion of the population – even within a high-risk area. In this case, the coverage proportion was not applied directly to the population but was adjusted downward to account for the fractional population.

$$Coverage_{full\ population} = Coverage\ data\ point * \frac{Campaign\ target\ population}{IHME\ National\ population}$$

$$Coverage_{high-risk\ population} = Coverage\ data\ point * \frac{Campaign\ target\ population}{IHME\ national\ population * high - risk\ proportion}$$

In addition, if coverage for a multi-year campaign was reported separately for separate years and subnational target populations, and these subgroups were intended to comprise the full country (or full high-risk area, for the high-risk analysis), the population scalars were squeezed so that they would sum to one. If a campaign was stated to be national, this adjustment was not applied, even if the campaign target population was not equal to the estimated IHME population. In these cases, the coverage data point was applied as stated from the Bwaka *et al.* or WHO source.

Estimating variance for data points

Variance for survey and administrative data points was calculated using the method described in Galles *et al.*, appendix section 2.4.1 and 2.4.2.[7] For the campaign data from Bwaka *et al.*, a sample size of 100 was assumed for

purposes of variance calculation. When adjustment coefficients were applied to adjust administrative data to the level of survey data, uncertainty was not additionally propagated, a limitation of the current study.

Bias adjustment for campaign data

To model the adjustment ratio using the CrossWalk package, first, we generate matched data pairs. All paired data were a set of alternative (administrative) and reference (survey) observations matched on location and year of campaign, extracted from Bwaka *et al.* or WHO WER reports, for data from year 2020 or prior. Out of thirty-eight location-years with data, twenty-one had both survey and administrative data available, and seventeen had only administrative data available; i.e., there were twenty-one pairs matched by location and year to generate the adjustment model, and seventeen data points that would need the adjustment applied to them. For each matched pair, we calculated the logit difference δ_i as:

$$\delta_i = \text{logit}(\text{alternative}_i) - \text{logit}(\text{reference}_i)$$

We used the CrossWalk package to run a meta-regression model that estimates how this logit difference varies by covariate values. We tested several covariates: socio-demographic index, DTP3 coverage, MCV1 coverage, and size of the population as potential predictors of the magnitude of the survey:administrative ratio. Our rationale for testing SDI was that higher SDI locations may have stronger health systems and/or capacity to track population sizes and vaccines delivered, and our rationale for DTP3/MCV1 coverage was that they could be used as proxies for the overall strength of immunisation systems. Our rationale for testing size of the population was that smaller campaigns might be easier to track, and therefore population might have an association with the magnitude of the bias ratio. When we looked at the association between each predictor and the ratio in a univariate regression, log(population size) was the only predictor that was significantly associated.

Therefore, we ran a mixed effects model, using natural log of the size of the target population as a fixed effect, with random effects on country. The model is:

$$\hat{\delta}_i = \beta_0 + \beta_1 \log(target) + u_j + \epsilon_i$$

where $\hat{\delta}_i$ is the modelled logit difference, β_0 is the intercept, $\beta_1$is the coefficient on log(target), u_j are random effects by country, and ϵ_i is the error term.

To obtain the estimated unbiased value for an administrative data point, we applied the predicted adjustment factor by subtracting it from the original observation in logit space

$$\mu_i' = logit^{-1}(logit(\mu_i) - \hat{\delta}_i)$$

where μ_i' is the estimated unbiased value and μ_i is the original value.

Bias adjustment for routine immunisation data

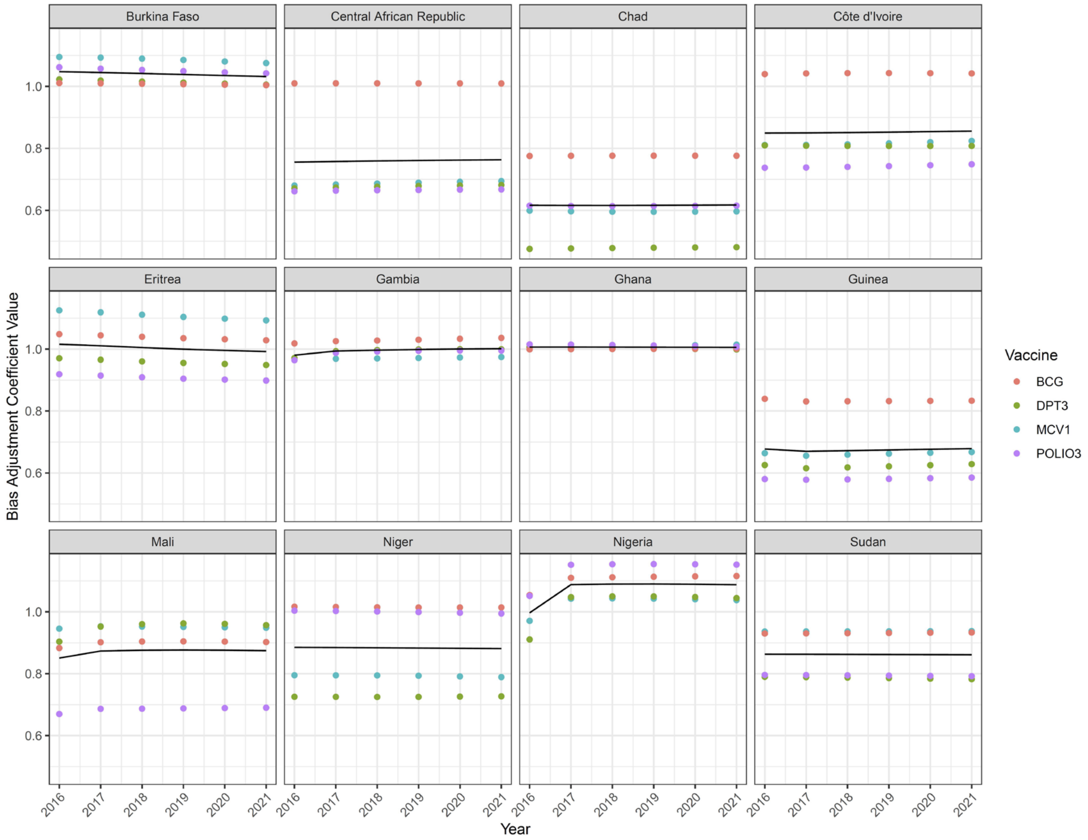

Figure S1: Survey:administrative coefficients used to adjust administrative for select vaccines up to the level of survey. Coefficients are sourced from the Global Burden of Disease study, for country-years with routine infant MenA immunisation in the meningitis belt. Points show the single vaccine adjustment coefficient for each vaccine, and the black line shows the average of the four coefficients. The coefficient is defined as survey:administrative, so this coefficient is multiplied by administrative data points to produce a value adjusted to the level of survey.

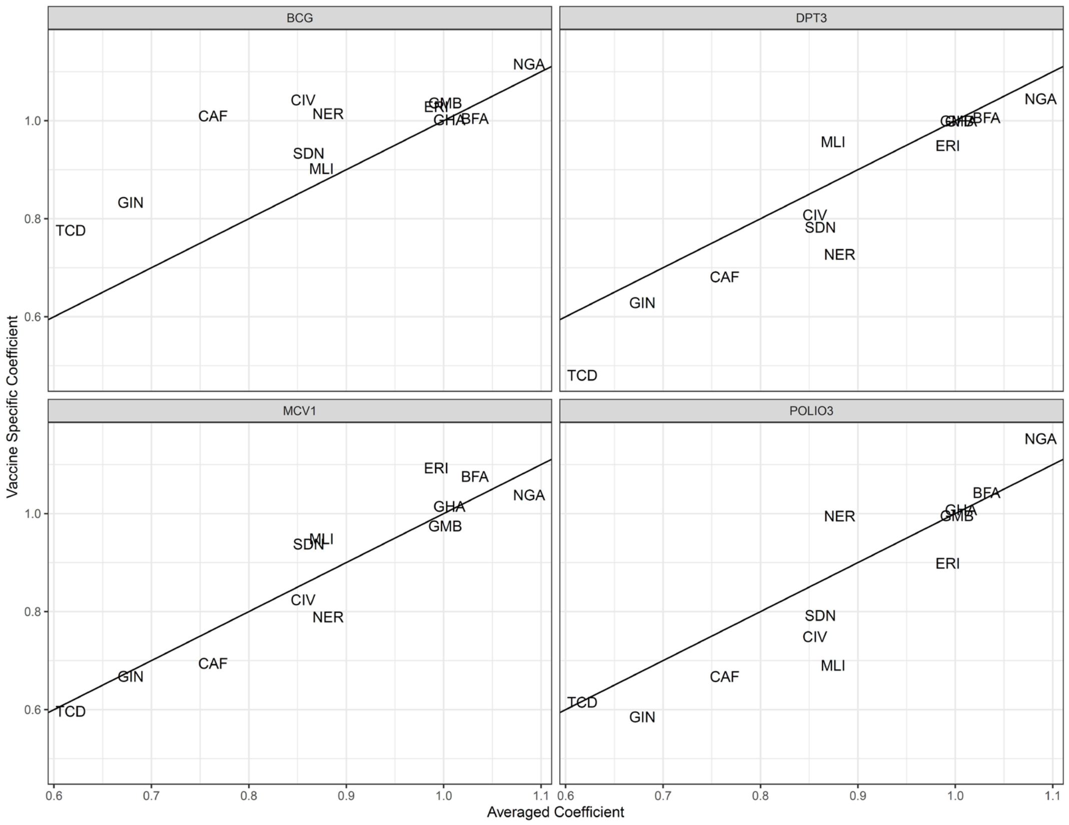

Figure S2: Comparison of survey:administrative bias adjustment coefficients for single vaccines to the averaged coefficient for 2021, which is used in routine MenA vaccine coverage adjustment.

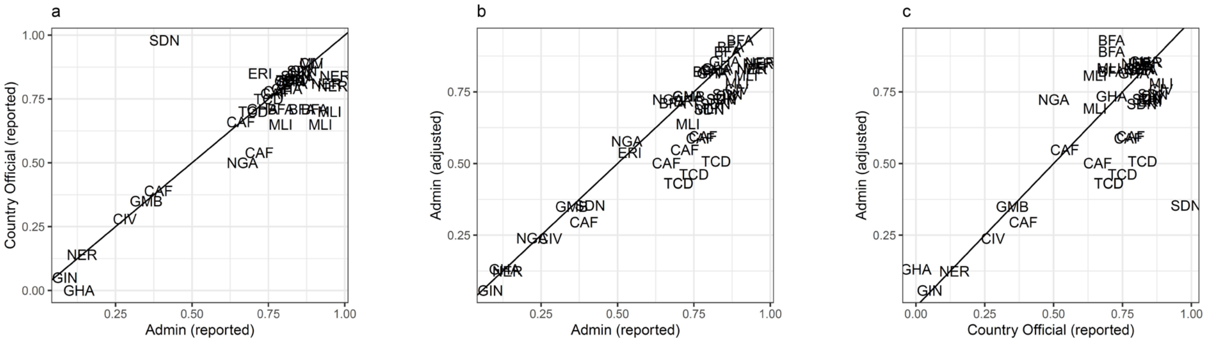

Figure S3: Comparison of country official vs. admin data reported by countries to the WHO immunisation data portal through the WHO/UNICEF Joint Reporting Form on Immunization (JRF) from 2016 to 2021 (a), adjusted admin to admin reported via JRF (b), and adjusted admin to country official reported via JRF (c).

Data input	Reference or alternative case definition	Gamma	Coefficient	Beta Coefficient, Logit (95% UI)*	Adjustment factor**
Survey	Ref	0.42		---	---
Administrative	Alt		Intercept	5.47 (-2.69, 13.64)	238

			Log_target	-0.38 (-0.9, 0.14)<br>-0.34 (-0.93, 0.24)	0.68

Table S2: Bias adjustment factors for campaign MenA vaccine coverage

*Bias adjustments can be interpreted as the factor the alternative case definition is adjusted by to reflect what it would have been had it been measured using the reference case definition. If the logit beta coefficient is negative, then the alternative is adjusted up to the reference. If the logit beta coefficient is positive, then the alternative is adjusted down to the reference.
**The adjustment factor column is the exponentiated beta coefficient. For logit beta coefficients, this is the relative odds between the two case definitions.

Adherence to reporting guidelines

GATHER
Guidelines for Accurate and Transparent Health Estimates Reporting

Item #	Checklist item	Reported in section(s)
**Objectives and funding**		
**1**	Define the indicator(s), populations (including age, sex, and geographic entities), and time period(s) for which estimates were made.	Introduction<br>Methods
**2**	List the funding sources for the work.	Funding
**Data Inputs**		
For all data inputs from multiple sources that are synthesized as part of the study:		
**3**	Describe how the data were identified and how the data were accessed.	Methods (Data sources)<br>Supplementary material (Data)
**4**	Specify the inclusion and exclusion criteria. Identify all ad-hoc exclusions.	Supplementary material (Data)
**5**	Provide information on all included data sources and their main characteristics. For each data source used, report reference information or contact name/institution, population represented, data collection method, year(s) of data collection, sex and age range, diagnostic criteria or measurement method, and sample size, as relevant.	Metadata for all data sources can be freely accessed in the Global Health Data Exchange (https://ghdx.healthdata.org/record/ihme-data/sub-saharan-africa-menafrivac-estimates-2010-2021)
**6**	Identify and describe any categories of input data that have potentially important biases (e.g., based on characteristics listed in item 5).	Methods (Administrative bias adjustment: campaign data; Administrative bias adjustment: RI data)<br>Supplementary material (Methods)
For data inputs that contribute to the analysis but were not synthesized as part of the study:		
**7**	Describe and give sources for any other data inputs.	Methods (Data sources)

		Supplementary material (Data)
For all data inputs:		
**8**	Provide all data inputs in a file format from which data can be efficiently extracted (e.g., a spreadsheet rather than a PDF), including all relevant meta-data listed in item 5. For any data inputs that cannot be shared because of ethical or legal reasons, such as third-party ownership, provide a contact name or the name of the institution that retains the right to the data.	Metadata for all data sources can be freely accessed in the Global Health Data Exchange (http://ghdx.healthdata.org/record/ihme-data/sub-saharan-africa-menafrivac-estimates-2010-2021)
**Data analysis**		
**9**	Provide a conceptual overview of the data analysis method. A diagram may be helpful.	Figure 1
**10**	Provide a detailed description of all steps of the analysis, including mathematical formulae. This description should cover, as relevant, data cleaning, data pre-processing, data adjustments and weighting of data sources, and mathematical or statistical model(s).	Methods<br>Supplementary Material (Methods)
**11**	Describe how candidate models were evaluated and how the final model(s) were selected.	NA
**12**	Provide the results of an evaluation of model performance, if done, as well as the results of any relevant sensitivity analysis.	NA
**13**	Describe methods for calculating uncertainty of the estimates. State which sources of uncertainty were, and were not, accounted for in the uncertainty analysis.	Methods (Routine immunisation coverage estimation; Overall vaccination coverage estimation: routine plus campaigns)<br>Supplementary Material (Methods: Estimating variance for data points)
**14**	State how analytic or statistical source code used to generate estimates can be accessed.	Public repository available at: https://github.com/ihmeuw/mena_coverage
**Results and Discussion**		
**15**	Provide published estimates in a file format from which data can be efficiently extracted.	CSV of results can be freely accessed in the Global Health Data Exchange (http://ghdx.healthdata.org/record/ihme-data/sub-saharan-africa-menafrivac-estimates-2010-2021)
**16**	Report a quantitative measure of the uncertainty of the estimates (e.g. uncertainty intervals).	CSV of results can be freely accessed in the Global Health Data Exchange (http://ghdx.healthdata.org/record/ihme-data/sub-saharan-africa-menafrivac-estimates-2010-2021)

17	Interpret results in light of existing evidence. If updating a previous set of estimates, describe the reasons for changes in estimates.	Results<br><br>Discussion
18	Discuss limitations of the estimates. Include a discussion of any modelling assumptions or data limitations that affect interpretation of the estimates.	Discussion

Table S3: Checklist of information from GATHER included in the current report.

Results

Supplemental Figures

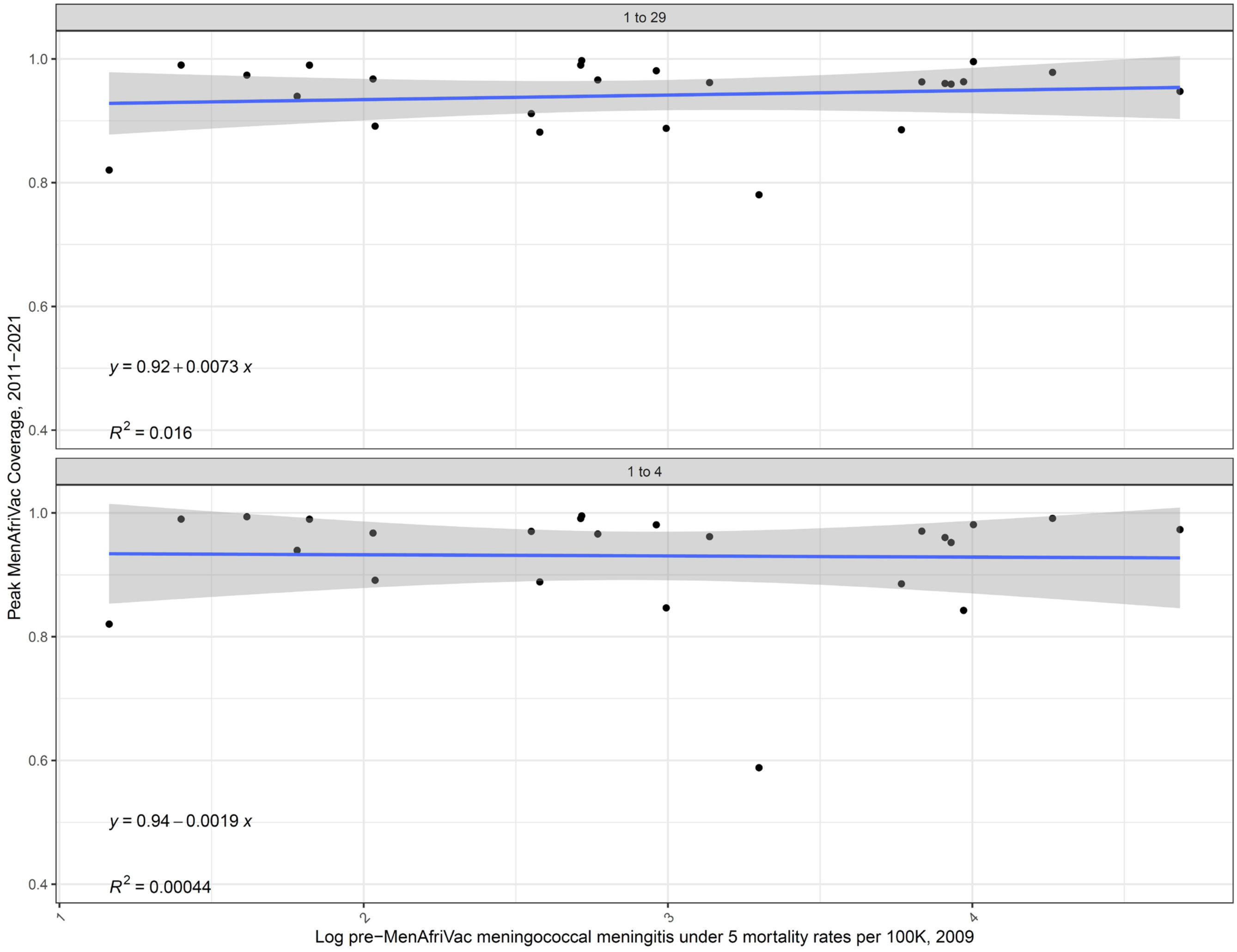

Figure S4: Relationship between the natural log of pre-MACV (year 2009) mortality rate in children under 5 due to meningococcal meningitis and peak MACV coverage in high-risk populations for ages 1-4.

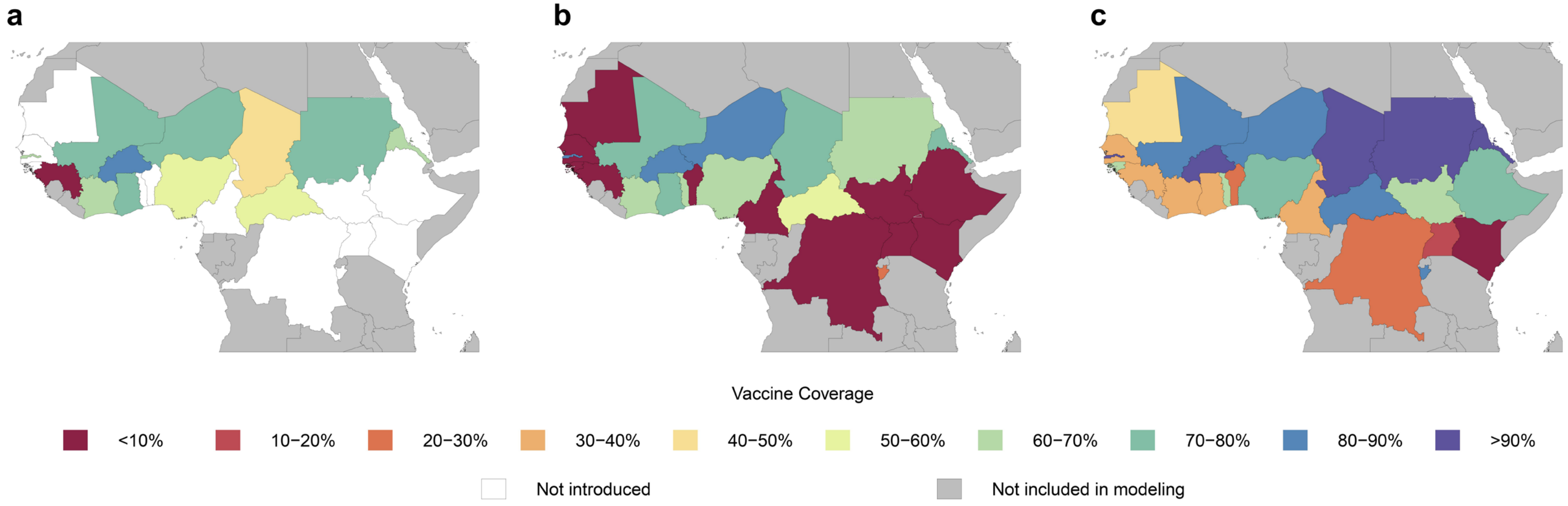

Figure S5: Coverage estimates for full country populations for the meningitis belt, at year end 2021. (a) Shows the routine immunisation values for the target age, which varies by country from 9 to 18 months. (b) Shows combined coverage estimates for mass campaigns, catch-up campaigns, and routine immunisation coverage combined for ages 1–4. (c) Shows combined coverage estimates for ages 1–29. Analogous to main text figure 3.

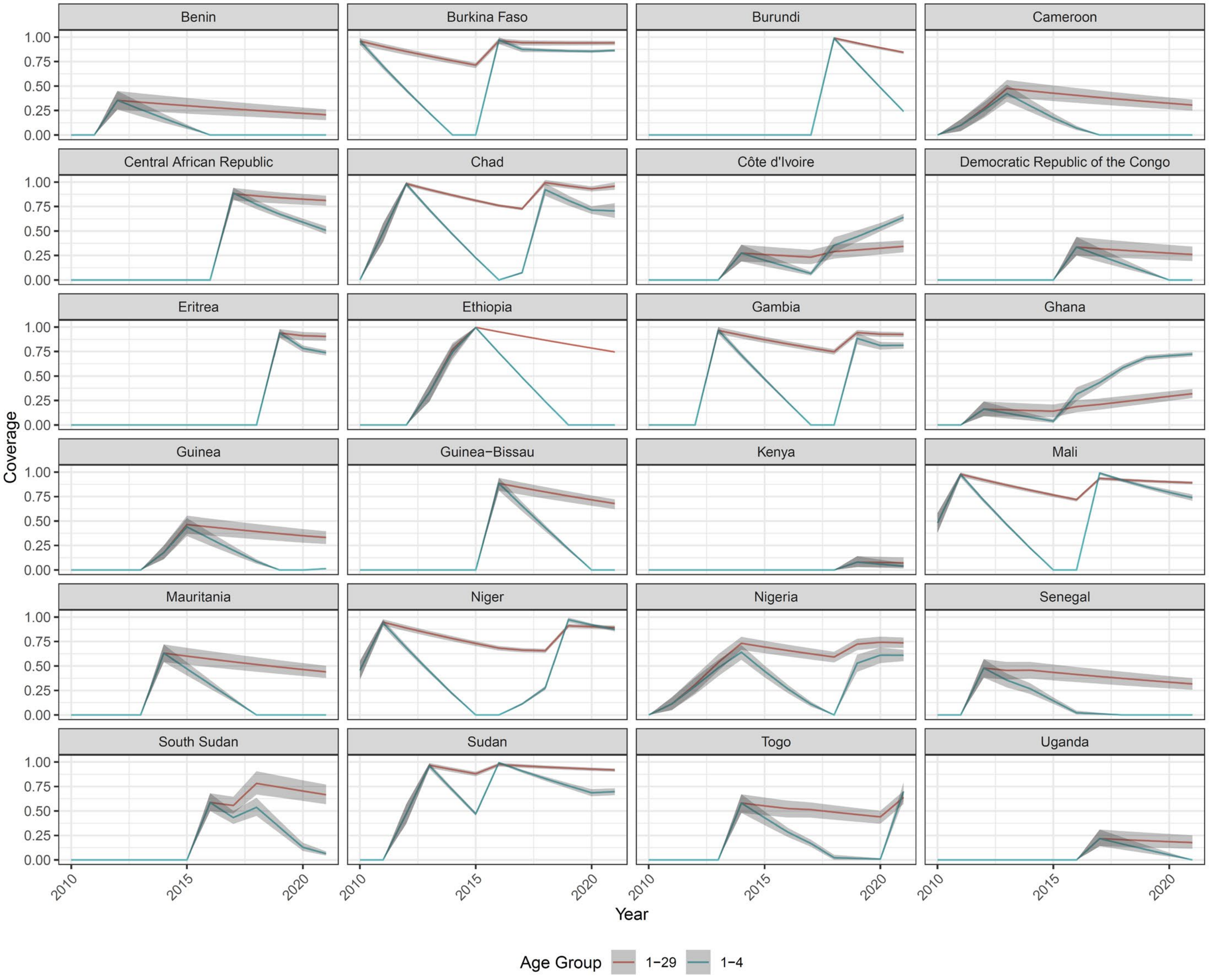

Figure S6: Coverage estimates for full country populations for the meningitis belt, for 1–29 and 1–4 age groups, from 2010 to 2021. Year-end coverage values shown for each year. Analogous to main text figure 4.

Supplemental Table

Location	Sex	Age	Year	Analysis	Mean	Lower	Upper
**Benin**	Both	1–4	2010	high-risk	0.00%	0.00%	0.00%
**Benin**	Both	1–29	2010	high-risk	0.00%	0.00%	0.00%
**Benin**	Both	1–4	2011	high-risk	0.00%	0.00%	0.00%
**Benin**	Both	1–29	2011	high-risk	0.00%	0.00%	0.00%
**Benin**	Both	1–4	2012	high-risk	96.03%	92.00%	99.00%
**Benin**	Both	1–29	2012	high-risk	96.03%	92.00%	99.00%
**Benin**	Both	1–4	2013	high-risk	70.62%	67.66%	72.81%
**Benin**	Both	1–29	2013	high-risk	90.75%	86.94%	93.55%
**Benin**	Both	1–4	2014	high-risk	46.31%	44.36%	47.74%
**Benin**	Both	1–29	2014	high-risk	85.75%	82.15%	88.40%
**Benin**	Both	1–4	2015	high-risk	22.81%	21.85%	23.51%
**Benin**	Both	1–29	2015	high-risk	80.98%	77.58%	83.48%
**Benin**	Both	1–4	2016	high-risk	0.00%	0.00%	0.00%
**Benin**	Both	1–29	2016	high-risk	76.41%	73.20%	78.77%
**Benin**	Both	1–4	2017	high-risk	0.00%	0.00%	0.00%
**Benin**	Both	1–29	2017	high-risk	72.03%	69.00%	74.25%
**Benin**	Both	1–4	2018	high-risk	0.00%	0.00%	0.00%
**Benin**	Both	1–29	2018	high-risk	67.81%	64.97%	69.91%
**Benin**	Both	1–4	2019	high-risk	0.00%	0.00%	0.00%
**Benin**	Both	1–29	2019	high-risk	63.76%	61.09%	65.74%
**Benin**	Both	1–4	2020	high-risk	0.00%	0.00%	0.00%
**Benin**	Both	1–29	2020	high-risk	59.87%	57.36%	61.72%
**Benin**	Both	1–4	2021	high-risk	0.00%	0.00%	0.00%
**Benin**	Both	1–29	2021	high-risk	56.14%	53.78%	57.88%
**Burkina Faso**	Both	1–4	2010	high-risk	95.95%	92.00%	99.00%
**Burkina Faso**	Both	1–29	2010	high-risk	95.95%	92.00%	99.00%
**Burkina Faso**	Both	1–4	2011	high-risk	70.22%	67.33%	72.46%
**Burkina Faso**	Both	1–29	2011	high-risk	90.51%	86.78%	93.38%
**Burkina Faso**	Both	1–4	2012	high-risk	45.88%	43.99%	47.34%
**Burkina Faso**	Both	1–29	2012	high-risk	85.39%	81.88%	88.11%
**Burkina Faso**	Both	1–4	2013	high-risk	22.52%	21.59%	23.24%
**Burkina Faso**	Both	1–29	2013	high-risk	80.52%	77.21%	83.08%
**Burkina Faso**	Both	1–4	2014	high-risk	0.00%	0.00%	0.00%
**Burkina Faso**	Both	1–29	2014	high-risk	75.86%	72.74%	78.27%
**Burkina Faso**	Both	1–4	2015	high-risk	0.00%	0.00%	0.00%
**Burkina Faso**	Both	1–29	2015	high-risk	71.37%	68.43%	73.64%
**Burkina Faso**	Both	1–4	2016	high-risk	97.05%	94.00%	100.00%
**Burkina Faso**	Both	1–29	2016	high-risk	96.28%	93.00%	99.00%
**Burkina Faso**	Both	1–4	2017	high-risk	87.59%	85.13%	89.59%
**Burkina Faso**	Both	1–29	2017	high-risk	94.36%	91.33%	96.87%

Location	Sex	Age	Year	Analysis	Mean	Lower	Upper
**Burkina Faso**	Both	1–4	2018	high-risk	86.63%	84.90%	88.13%
**Burkina Faso**	Both	1–29	2018	high-risk	94.21%	91.40%	96.56%
**Burkina Faso**	Both	1–4	2019	high-risk	85.88%	84.40%	87.17%
**Burkina Faso**	Both	1–29	2019	high-risk	94.11%	91.48%	96.33%
**Burkina Faso**	Both	1–4	2020	high-risk	85.53%	83.99%	86.87%
**Burkina Faso**	Both	1–29	2020	high-risk	94.11%	91.64%	96.19%
**Burkina Faso**	Both	1–4	2021	high-risk	86.42%	85.09%	87.61%
**Burkina Faso**	Both	1–29	2021	high-risk	94.14%	91.81%	96.10%
**Burundi**	Both	1–4	2010	high-risk	0.00%	0.00%	0.00%
**Burundi**	Both	1–29	2010	high-risk	0.00%	0.00%	0.00%
**Burundi**	Both	1–4	2011	high-risk	0.00%	0.00%	0.00%
**Burundi**	Both	1–29	2011	high-risk	0.00%	0.00%	0.00%
**Burundi**	Both	1–4	2012	high-risk	0.00%	0.00%	0.00%
**Burundi**	Both	1–29	2012	high-risk	0.00%	0.00%	0.00%
**Burundi**	Both	1–4	2013	high-risk	0.00%	0.00%	0.00%
**Burundi**	Both	1–29	2013	high-risk	0.00%	0.00%	0.00%
**Burundi**	Both	1–4	2014	high-risk	0.00%	0.00%	0.00%
**Burundi**	Both	1–29	2014	high-risk	0.00%	0.00%	0.00%
**Burundi**	Both	1–4	2015	high-risk	0.00%	0.00%	0.00%
**Burundi**	Both	1–29	2015	high-risk	0.00%	0.00%	0.00%
**Burundi**	Both	1–4	2016	high-risk	0.00%	0.00%	0.00%
**Burundi**	Both	1–29	2016	high-risk	0.00%	0.00%	0.00%
**Burundi**	Both	1–4	2017	high-risk	0.00%	0.00%	0.00%
**Burundi**	Both	1–29	2017	high-risk	0.00%	0.00%	0.00%
**Burundi**	Both	1–4	2018	high-risk	98.99%	97.00%	100.00%
**Burundi**	Both	1–29	2018	high-risk	98.99%	97.00%	100.00%
**Burundi**	Both	1–4	2019	high-risk	73.44%	71.96%	74.19%
**Burundi**	Both	1–29	2019	high-risk	93.95%	92.06%	94.91%
**Burundi**	Both	1–4	2020	high-risk	48.52%	47.55%	49.02%
**Burundi**	Both	1–29	2020	high-risk	89.14%	87.34%	90.04%
**Burundi**	Both	1–4	2021	high-risk	24.08%	23.60%	24.33%
**Burundi**	Both	1–29	2021	high-risk	84.52%	82.82%	85.38%
**Cameroon**	Both	1–4	2010	high-risk	0.00%	0.00%	0.00%
**Cameroon**	Both	1–29	2010	high-risk	0.00%	0.00%	0.00%
**Cameroon**	Both	1–4	2011	high-risk	24.35%	16.00%	33.00%
**Cameroon**	Both	1–29	2011	high-risk	24.35%	16.00%	33.00%
**Cameroon**	Both	1–4	2012	high-risk	58.28%	49.71%	66.81%
**Cameroon**	Both	1–29	2012	high-risk	60.90%	52.71%	69.54%
**Cameroon**	Both	1–4	2013	high-risk	84.66%	79.05%	89.85%
**Cameroon**	Both	1–29	2013	high-risk	88.77%	84.15%	92.84%
**Cameroon**	Both	1–4	2014	high-risk	61.33%	56.79%	65.49%
**Cameroon**	Both	1–29	2014	high-risk	84.16%	79.78%	88.02%

Location	Sex	Age	Year	Analysis	Mean	Lower	Upper
**Cameroon**	Both	1–4	2015	high-risk	38.84%	35.37%	42.02%
**Cameroon**	Both	1–29	2015	high-risk	79.80%	75.63%	83.46%
**Cameroon**	Both	1–4	2016	high-risk	17.69%	15.65%	19.74%
**Cameroon**	Both	1–29	2016	high-risk	75.63%	71.67%	79.11%
**Cameroon**	Both	1–4	2017	high-risk	0.00%	0.00%	0.00%
**Cameroon**	Both	1–29	2017	high-risk	71.64%	67.88%	74.95%
**Cameroon**	Both	1–4	2018	high-risk	0.00%	0.00%	0.00%
**Cameroon**	Both	1–29	2018	high-risk	67.82%	64.26%	70.96%
**Cameroon**	Both	1–4	2019	high-risk	0.00%	0.00%	0.00%
**Cameroon**	Both	1–29	2019	high-risk	64.16%	60.78%	67.14%
**Cameroon**	Both	1–4	2020	high-risk	0.00%	0.00%	0.00%
**Cameroon**	Both	1–29	2020	high-risk	60.64%	57.43%	63.47%
**Cameroon**	Both	1–4	2021	high-risk	0.00%	0.00%	0.00%
**Cameroon**	Both	1–29	2021	high-risk	57.27%	54.22%	59.94%
**Central African Republic**	Both	1–4	2010	high-risk	0.00%	0.00%	0.00%
**Central African Republic**	Both	1–29	2010	high-risk	0.00%	0.00%	0.00%
**Central African Republic**	Both	1–4	2011	high-risk	0.00%	0.00%	0.00%
**Central African Republic**	Both	1–29	2011	high-risk	0.00%	0.00%	0.00%
**Central African Republic**	Both	1–4	2012	high-risk	0.00%	0.00%	0.00%
**Central African Republic**	Both	1–29	2012	high-risk	0.00%	0.00%	0.00%
**Central African Republic**	Both	1–4	2013	high-risk	0.00%	0.00%	0.00%
**Central African Republic**	Both	1–29	2013	high-risk	0.00%	0.00%	0.00%
**Central African Republic**	Both	1–4	2014	high-risk	0.00%	0.00%	0.00%
**Central African Republic**	Both	1–29	2014	high-risk	0.00%	0.00%	0.00%
**Central African Republic**	Both	1–4	2015	high-risk	0.00%	0.00%	0.00%
**Central African Republic**	Both	1–29	2015	high-risk	0.00%	0.00%	0.00%
**Central African Republic**	Both	1–4	2016	high-risk	0.00%	0.00%	0.00%
**Central African Republic**	Both	1–29	2016	high-risk	0.00%	0.00%	0.00%
**Central African Republic**	Both	1–4	2017	high-risk	88.86%	82.44%	94.42%
**Central African Republic**	Both	1–29	2017	high-risk	88.16%	81.28%	94.08%
**Central African Republic**	Both	1–4	2018	high-risk	77.16%	72.71%	81.34%
**Central African Republic**	Both	1–29	2018	high-risk	85.93%	79.48%	91.49%
**Central African Republic**	Both	1–4	2019	high-risk	67.28%	63.85%	70.83%
**Central African Republic**	Both	1–29	2019	high-risk	84.09%	78.02%	89.31%
**Central African Republic**	Both	1–4	2020	high-risk	59.05%	55.65%	62.57%
**Central African Republic**	Both	1–29	2020	high-risk	82.60%	76.93%	87.54%
**Central African Republic**	Both	1–4	2021	high-risk	50.72%	46.88%	54.94%
**Central African Republic**	Both	1–29	2021	high-risk	81.26%	75.88%	86.05%
**Chad**	Both	1–4	2010	high-risk	0.00%	0.00%	0.00%
**Chad**	Both	1–29	2010	high-risk	0.00%	0.00%	0.00%
**Chad**	Both	1–4	2011	high-risk	48.56%	39.00%	59.00%
**Chad**	Both	1–29	2011	high-risk	48.56%	39.00%	59.00%

Location	Sex	Age	Year	Analysis	Mean	Lower	Upper
**Chad**	Both	1–4	2012	high-risk	98.11%	95.75%	100.00%
**Chad**	Both	1–29	2012	high-risk	98.40%	96.28%	100.00%
**Chad**	Both	1–4	2013	high-risk	71.71%	69.86%	73.18%
**Chad**	Both	1–29	2013	high-risk	92.35%	90.36%	93.85%
**Chad**	Both	1–4	2014	high-risk	46.72%	45.39%	47.79%
**Chad**	Both	1–29	2014	high-risk	86.67%	84.80%	88.08%
**Chad**	Both	1–4	2015	high-risk	22.76%	21.80%	23.44%
**Chad**	Both	1–29	2015	high-risk	81.27%	79.52%	82.59%
**Chad**	Both	1–4	2016	high-risk	0.00%	0.00%	0.00%
**Chad**	Both	1–29	2016	high-risk	76.12%	74.47%	77.36%
**Chad**	Both	1–4	2017	high-risk	7.43%	6.59%	8.30%
**Chad**	Both	1–29	2017	high-risk	72.87%	71.36%	74.03%
**Chad**	Both	1–4	2018	high-risk	92.50%	85.54%	98.86%
**Chad**	Both	1–29	2018	high-risk	99.55%	96.63%	102.32%
**Chad**	Both	1–4	2019	high-risk	81.34%	76.02%	86.28%
**Chad**	Both	1–29	2019	high-risk	96.21%	93.43%	98.94%
**Chad**	Both	1–4	2020	high-risk	71.54%	67.45%	75.57%
**Chad**	Both	1–29	2020	high-risk	93.21%	90.50%	95.84%
**Chad**	Both	1–4	2021	high-risk	70.53%	63.84%	77.89%
**Chad**	Both	1–29	2021	high-risk	95.97%	92.54%	99.58%
**Côte d'Ivoire**	Both	1–4	2010	high-risk	0.00%	0.00%	0.00%
**Côte d'Ivoire**	Both	1–29	2010	high-risk	0.00%	0.00%	0.00%
**Côte d'Ivoire**	Both	1–4	2011	high-risk	0.00%	0.00%	0.00%
**Côte d'Ivoire**	Both	1–29	2011	high-risk	0.00%	0.00%	0.00%
**Côte d'Ivoire**	Both	1–4	2012	high-risk	0.00%	0.00%	0.00%
**Côte d'Ivoire**	Both	1–29	2012	high-risk	0.00%	0.00%	0.00%
**Côte d'Ivoire**	Both	1–4	2013	high-risk	0.00%	0.00%	0.00%
**Côte d'Ivoire**	Both	1–29	2013	high-risk	0.00%	0.00%	0.00%
**Côte d'Ivoire**	Both	1–4	2014	high-risk	98.98%	97.00%	100.00%
**Côte d'Ivoire**	Both	1–29	2014	high-risk	98.98%	97.00%	100.00%
**Côte d'Ivoire**	Both	1–4	2015	high-risk	73.04%	71.58%	73.79%
**Côte d'Ivoire**	Both	1–29	2015	high-risk	93.89%	92.02%	94.86%
**Côte d'Ivoire**	Both	1–4	2016	high-risk	48.04%	47.08%	48.53%
**Côte d'Ivoire**	Both	1–29	2016	high-risk	89.02%	87.24%	89.94%
**Côte d'Ivoire**	Both	1–4	2017	high-risk	23.73%	23.26%	23.97%
**Côte d'Ivoire**	Both	1–29	2017	high-risk	84.32%	82.63%	85.19%
**Côte d'Ivoire**	Both	1–4	2018	high-risk	99.10%	97.32%	100.00%
**Côte d'Ivoire**	Both	1–29	2018	high-risk	99.00%	97.24%	100.00%
**Côte d'Ivoire**	Both	1–4	2019	high-risk	89.59%	87.79%	91.11%
**Côte d'Ivoire**	Both	1–29	2019	high-risk	97.17%	95.44%	98.22%
**Côte d'Ivoire**	Both	1–4	2020	high-risk	81.41%	79.27%	83.68%
**Côte d'Ivoire**	Both	1–29	2020	high-risk	95.62%	93.94%	96.75%

Location	Sex	Age	Year	Analysis	Mean	Lower	Upper
**Côte d'Ivoire**	Both	1–4	2021	high-risk	73.98%	71.27%	76.96%
**Côte d'Ivoire**	Both	1–29	2021	high-risk	94.25%	92.59%	95.44%
**Democratic Republic of the Congo**	Both	1–4	2010	high-risk	0.00%	0.00%	0.00%
**Democratic Republic of the Congo**	Both	1–29	2010	high-risk	0.00%	0.00%	0.00%
**Democratic Republic of the Congo**	Both	1–4	2011	high-risk	0.00%	0.00%	0.00%
**Democratic Republic of the Congo**	Both	1–29	2011	high-risk	0.00%	0.00%	0.00%
**Democratic Republic of the Congo**	Both	1–4	2012	high-risk	0.00%	0.00%	0.00%
**Democratic Republic of the Congo**	Both	1–29	2012	high-risk	0.00%	0.00%	0.00%
**Democratic Republic of the Congo**	Both	1–4	2013	high-risk	0.00%	0.00%	0.00%
**Democratic Republic of the Congo**	Both	1–29	2013	high-risk	0.00%	0.00%	0.00%
**Democratic Republic of the Congo**	Both	1–4	2014	high-risk	0.00%	0.00%	0.00%
**Democratic Republic of the Congo**	Both	1–29	2014	high-risk	0.00%	0.00%	0.00%
**Democratic Republic of the Congo**	Both	1–4	2015	high-risk	0.00%	0.00%	0.00%
**Democratic Republic of the Congo**	Both	1–29	2015	high-risk	0.00%	0.00%	0.00%
**Democratic Republic of the Congo**	Both	1–4	2016	high-risk	99.01%	97.00%	100.00%
**Democratic Republic of the Congo**	Both	1–29	2016	high-risk	99.01%	97.00%	100.00%
**Democratic Republic of the Congo**	Both	1–4	2017	high-risk	73.48%	72.00%	74.22%
**Democratic Republic of the Congo**	Both	1–29	2017	high-risk	94.09%	92.18%	95.03%
**Democratic Republic of the Congo**	Both	1–4	2018	high-risk	48.61%	47.63%	49.10%
**Democratic Republic of the Congo**	Both	1–29	2018	high-risk	89.42%	87.61%	90.32%
**Democratic Republic of the Congo**	Both	1–4	2019	high-risk	24.17%	23.68%	24.41%
**Democratic Republic of the Congo**	Both	1–29	2019	high-risk	84.99%	83.26%	85.84%
**Democratic Republic of the Congo**	Both	1–4	2020	high-risk	0.00%	0.00%	0.00%
**Democratic Republic of the Congo**	Both	1–29	2020	high-risk	80.75%	79.11%	81.56%
**Democratic Republic of the Congo**	Both	1–4	2021	high-risk	0.00%	0.00%	0.00%
**Democratic Republic of the Congo**	Both	1–29	2021	high-risk	76.70%	75.15%	77.47%
**Eritrea**	Both	1–4	2010	high-risk	0.00%	0.00%	0.00%
**Eritrea**	Both	1–29	2010	high-risk	0.00%	0.00%	0.00%
**Eritrea**	Both	1–4	2011	high-risk	0.00%	0.00%	0.00%
**Eritrea**	Both	1–29	2011	high-risk	0.00%	0.00%	0.00%
**Eritrea**	Both	1–4	2012	high-risk	0.00%	0.00%	0.00%
**Eritrea**	Both	1–29	2012	high-risk	0.00%	0.00%	0.00%
**Eritrea**	Both	1–4	2013	high-risk	0.00%	0.00%	0.00%
**Eritrea**	Both	1–29	2013	high-risk	0.00%	0.00%	0.00%
**Eritrea**	Both	1–4	2014	high-risk	0.00%	0.00%	0.00%
**Eritrea**	Both	1–29	2014	high-risk	0.00%	0.00%	0.00%
**Eritrea**	Both	1–4	2015	high-risk	0.00%	0.00%	0.00%
**Eritrea**	Both	1–29	2015	high-risk	0.00%	0.00%	0.00%
**Eritrea**	Both	1–4	2016	high-risk	0.00%	0.00%	0.00%
**Eritrea**	Both	1–29	2016	high-risk	0.00%	0.00%	0.00%
**Eritrea**	Both	1–4	2017	high-risk	0.00%	0.00%	0.00%
**Eritrea**	Both	1–29	2017	high-risk	0.00%	0.00%	0.00%

Location	Sex	Age	Year	Analysis	Mean	Lower	Upper
**Eritrea**	Both	1–4	2018	high-risk	0.00%	0.00%	0.00%
**Eritrea**	Both	1–29	2018	high-risk	0.00%	0.00%	0.00%
**Eritrea**	Both	1–4	2019	high-risk	93.96%	89.00%	98.00%
**Eritrea**	Both	1–29	2019	high-risk	93.96%	89.00%	98.00%
**Eritrea**	Both	1–4	2020	high-risk	78.22%	74.76%	81.19%
**Eritrea**	Both	1–29	2020	high-risk	91.19%	86.51%	95.06%
**Eritrea**	Both	1–4	2021	high-risk	73.82%	71.28%	76.23%
**Eritrea**	Both	1–29	2021	high-risk	90.45%	85.89%	94.20%
**Ethiopia**	Both	1–4	2010	high-risk	0.00%	0.00%	0.00%
**Ethiopia**	Both	1–29	2010	high-risk	0.00%	0.00%	0.00%
**Ethiopia**	Both	1–4	2011	high-risk	0.00%	0.00%	0.00%
**Ethiopia**	Both	1–29	2011	high-risk	0.00%	0.00%	0.00%
**Ethiopia**	Both	1–4	2012	high-risk	0.00%	0.00%	0.00%
**Ethiopia**	Both	1–29	2012	high-risk	0.00%	0.00%	0.00%
**Ethiopia**	Both	1–4	2013	high-risk	33.26%	25.00%	42.00%
**Ethiopia**	Both	1–29	2013	high-risk	33.26%	25.00%	42.00%
**Ethiopia**	Both	1–4	2014	high-risk	74.91%	67.22%	81.88%
**Ethiopia**	Both	1–29	2014	high-risk	77.25%	69.56%	83.79%
**Ethiopia**	Both	1–4	2015	high-risk	99.53%	98.52%	100.00%
**Ethiopia**	Both	1–29	2015	high-risk	99.73%	99.07%	100.00%
**Ethiopia**	Both	1–4	2016	high-risk	73.80%	72.95%	74.21%
**Ethiopia**	Both	1–29	2016	high-risk	95.23%	94.60%	95.49%
**Ethiopia**	Both	1–4	2017	high-risk	48.62%	47.92%	48.96%
**Ethiopia**	Both	1–29	2017	high-risk	90.88%	90.28%	91.13%
**Ethiopia**	Both	1–4	2018	high-risk	23.97%	23.50%	24.22%
**Ethiopia**	Both	1–29	2018	high-risk	86.66%	86.09%	86.90%
**Ethiopia**	Both	1–4	2019	high-risk	0.00%	0.00%	0.00%
**Ethiopia**	Both	1–29	2019	high-risk	82.55%	82.00%	82.78%
**Ethiopia**	Both	1–4	2020	high-risk	0.00%	0.00%	0.00%
**Ethiopia**	Both	1–29	2020	high-risk	78.54%	78.02%	78.76%
**Ethiopia**	Both	1–4	2021	high-risk	0.00%	0.00%	0.00%
**Ethiopia**	Both	1–29	2021	high-risk	74.62%	74.11%	74.83%
**Gambia**	Both	1–4	2010	high-risk	0.00%	0.00%	0.00%
**Gambia**	Both	1–29	2010	high-risk	0.00%	0.00%	0.00%
**Gambia**	Both	1–4	2011	high-risk	0.00%	0.00%	0.00%
**Gambia**	Both	1–29	2011	high-risk	0.00%	0.00%	0.00%
**Gambia**	Both	1–4	2012	high-risk	0.00%	0.00%	0.00%
**Gambia**	Both	1–29	2012	high-risk	0.00%	0.00%	0.00%
**Gambia**	Both	1–4	2013	high-risk	96.60%	93.00%	100.00%
**Gambia**	Both	1–29	2013	high-risk	96.60%	93.00%	100.00%
**Gambia**	Both	1–4	2014	high-risk	71.33%	68.67%	73.84%
**Gambia**	Both	1–29	2014	high-risk	91.69%	88.27%	94.92%

Location	Sex	Age	Year	Analysis	Mean	Lower	Upper
**Gambia**	Both	1–4	2015	high-risk	47.00%	45.25%	48.65%
**Gambia**	Both	1–29	2015	high-risk	87.09%	83.85%	90.16%
**Gambia**	Both	1–4	2016	high-risk	23.29%	22.42%	24.11%
**Gambia**	Both	1–29	2016	high-risk	82.76%	79.68%	85.67%
**Gambia**	Both	1–4	2017	high-risk	0.00%	0.00%	0.00%
**Gambia**	Both	1–29	2017	high-risk	78.66%	75.73%	81.43%
**Gambia**	Both	1–4	2018	high-risk	0.00%	0.00%	0.00%
**Gambia**	Both	1–29	2018	high-risk	74.77%	71.98%	77.39%
**Gambia**	Both	1–4	2019	high-risk	88.27%	81.70%	93.91%
**Gambia**	Both	1–29	2019	high-risk	94.38%	91.15%	97.12%
**Gambia**	Both	1–4	2020	high-risk	81.04%	76.47%	85.12%
**Gambia**	Both	1–29	2020	high-risk	92.72%	89.60%	95.32%
**Gambia**	Both	1–4	2021	high-risk	81.30%	78.14%	84.41%
**Gambia**	Both	1–29	2021	high-risk	92.41%	89.45%	94.90%
**Ghana**	Both	1–4	2010	high-risk	0.00%	0.00%	0.00%
**Ghana**	Both	1–29	2010	high-risk	0.00%	0.00%	0.00%
**Ghana**	Both	1–4	2011	high-risk	0.00%	0.00%	0.00%
**Ghana**	Both	1–29	2011	high-risk	0.00%	0.00%	0.00%
**Ghana**	Both	1–4	2012	high-risk	89.94%	84.00%	95.02%
**Ghana**	Both	1–29	2012	high-risk	89.94%	84.00%	95.02%
**Ghana**	Both	1–4	2013	high-risk	66.65%	62.24%	70.41%
**Ghana**	Both	1–29	2013	high-risk	85.88%	80.21%	90.74%
**Ghana**	Both	1–4	2014	high-risk	43.98%	41.07%	46.46%
**Ghana**	Both	1–29	2014	high-risk	81.96%	76.54%	86.59%
**Ghana**	Both	1–4	2015	high-risk	21.79%	20.35%	23.02%
**Ghana**	Both	1–29	2015	high-risk	78.14%	72.98%	82.55%
**Ghana**	Both	1–4	2016	high-risk	97.02%	93.91%	100.00%
**Ghana**	Both	1–29	2016	high-risk	91.16%	85.79%	95.86%
**Ghana**	Both	1–4	2017	high-risk	81.17%	79.08%	82.80%
**Ghana**	Both	1–29	2017	high-risk	88.71%	83.66%	93.11%
**Ghana**	Both	1–4	2018	high-risk	77.54%	75.92%	79.01%
**Ghana**	Both	1–29	2018	high-risk	88.36%	83.67%	92.52%
**Ghana**	Both	1–4	2019	high-risk	74.20%	72.19%	75.99%
**Ghana**	Both	1–29	2019	high-risk	88.11%	83.60%	92.05%
**Ghana**	Both	1–4	2020	high-risk	70.73%	68.28%	72.98%
**Ghana**	Both	1–29	2020	high-risk	87.90%	83.62%	91.62%
**Ghana**	Both	1–4	2021	high-risk	72.36%	70.03%	74.50%
**Ghana**	Both	1–29	2021	high-risk	87.75%	83.70%	91.30%
**Guinea**	Both	1–4	2010	high-risk	0.00%	0.00%	0.00%
**Guinea**	Both	1–29	2010	high-risk	0.00%	0.00%	0.00%
**Guinea**	Both	1–4	2011	high-risk	0.00%	0.00%	0.00%
**Guinea**	Both	1–29	2011	high-risk	0.00%	0.00%	0.00%

Location	Sex	Age	Year	Analysis	Mean	Lower	Upper
**Guinea**	Both	1–4	2012	high-risk	0.00%	0.00%	0.00%
**Guinea**	Both	1–29	2012	high-risk	0.00%	0.00%	0.00%
**Guinea**	Both	1–4	2013	high-risk	0.00%	0.00%	0.00%
**Guinea**	Both	1–29	2013	high-risk	0.00%	0.00%	0.00%
**Guinea**	Both	1–4	2014	high-risk	46.37%	36.00%	56.00%
**Guinea**	Both	1–29	2014	high-risk	46.37%	36.00%	56.00%
**Guinea**	Both	1–4	2015	high-risk	95.23%	91.61%	98.13%
**Guinea**	Both	1–29	2015	high-risk	95.92%	92.65%	98.45%
**Guinea**	Both	1–4	2016	high-risk	70.18%	67.34%	72.44%
**Guinea**	Both	1–29	2016	high-risk	90.80%	87.71%	93.19%
**Guinea**	Both	1–4	2017	high-risk	46.00%	43.93%	47.64%
**Guinea**	Both	1–29	2017	high-risk	85.93%	82.99%	88.19%
**Guinea**	Both	1–4	2018	high-risk	22.38%	20.99%	23.40%
**Guinea**	Both	1–29	2018	high-risk	81.24%	78.47%	83.38%
**Guinea**	Both	1–4	2019	high-risk	0.00%	0.00%	0.00%
**Guinea**	Both	1–29	2019	high-risk	76.73%	74.10%	78.75%
**Guinea**	Both	1–4	2020	high-risk	0.00%	0.00%	0.00%
**Guinea**	Both	1–29	2020	high-risk	72.36%	69.88%	74.27%
**Guinea**	Both	1–4	2021	high-risk	1.32%	1.10%	1.57%
**Guinea**	Both	1–29	2021	high-risk	68.40%	66.07%	70.16%
**Guinea-Bissau**	Both	1–4	2010	high-risk	0.00%	0.00%	0.00%
**Guinea-Bissau**	Both	1–29	2010	high-risk	0.00%	0.00%	0.00%
**Guinea-Bissau**	Both	1–4	2011	high-risk	0.00%	0.00%	0.00%
**Guinea-Bissau**	Both	1–29	2011	high-risk	0.00%	0.00%	0.00%
**Guinea-Bissau**	Both	1–4	2012	high-risk	0.00%	0.00%	0.00%
**Guinea-Bissau**	Both	1–29	2012	high-risk	0.00%	0.00%	0.00%
**Guinea-Bissau**	Both	1–4	2013	high-risk	0.00%	0.00%	0.00%
**Guinea-Bissau**	Both	1–29	2013	high-risk	0.00%	0.00%	0.00%
**Guinea-Bissau**	Both	1–4	2014	high-risk	0.00%	0.00%	0.00%
**Guinea-Bissau**	Both	1–29	2014	high-risk	0.00%	0.00%	0.00%
**Guinea-Bissau**	Both	1–4	2015	high-risk	0.00%	0.00%	0.00%
**Guinea-Bissau**	Both	1–29	2015	high-risk	0.00%	0.00%	0.00%
**Guinea-Bissau**	Both	1–4	2016	high-risk	88.55%	82.00%	94.00%
**Guinea-Bissau**	Both	1–29	2016	high-risk	88.55%	82.00%	94.00%
**Guinea-Bissau**	Both	1–4	2017	high-risk	65.49%	60.65%	69.52%
**Guinea-Bissau**	Both	1–29	2017	high-risk	84.05%	77.83%	89.22%
**Guinea-Bissau**	Both	1–4	2018	high-risk	43.18%	39.99%	45.84%
**Guinea-Bissau**	Both	1–29	2018	high-risk	79.76%	73.86%	84.67%
**Guinea-Bissau**	Both	1–4	2019	high-risk	21.40%	19.82%	22.72%
**Guinea-Bissau**	Both	1–29	2019	high-risk	75.65%	70.06%	80.31%
**Guinea-Bissau**	Both	1–4	2020	high-risk	0.00%	0.00%	0.00%
**Guinea-Bissau**	Both	1–29	2020	high-risk	71.71%	66.41%	76.12%

Location	Sex	Age	Year	Analysis	Mean	Lower	Upper
**Guinea-Bissau**	Both	1–4	2021	high-risk	0.00%	0.00%	0.00%
**Guinea-Bissau**	Both	1–29	2021	high-risk	67.93%	62.90%	72.11%
**Kenya**	Both	1–4	2010	high-risk	0.00%	0.00%	0.00%
**Kenya**	Both	1–29	2010	high-risk	0.00%	0.00%	0.00%
**Kenya**	Both	1–4	2011	high-risk	0.00%	0.00%	0.00%
**Kenya**	Both	1–29	2011	high-risk	0.00%	0.00%	0.00%
**Kenya**	Both	1–4	2012	high-risk	0.00%	0.00%	0.00%
**Kenya**	Both	1–29	2012	high-risk	0.00%	0.00%	0.00%
**Kenya**	Both	1–4	2013	high-risk	0.00%	0.00%	0.00%
**Kenya**	Both	1–29	2013	high-risk	0.00%	0.00%	0.00%
**Kenya**	Both	1–4	2014	high-risk	0.00%	0.00%	0.00%
**Kenya**	Both	1–29	2014	high-risk	0.00%	0.00%	0.00%
**Kenya**	Both	1–4	2015	high-risk	0.00%	0.00%	0.00%
**Kenya**	Both	1–29	2015	high-risk	0.00%	0.00%	0.00%
**Kenya**	Both	1–4	2016	high-risk	0.00%	0.00%	0.00%
**Kenya**	Both	1–29	2016	high-risk	0.00%	0.00%	0.00%
**Kenya**	Both	1–4	2017	high-risk	0.00%	0.00%	0.00%
**Kenya**	Both	1–29	2017	high-risk	0.00%	0.00%	0.00%
**Kenya**	Both	1–4	2018	high-risk	0.00%	0.00%	0.00%
**Kenya**	Both	1–29	2018	high-risk	0.00%	0.00%	0.00%
**Kenya**	Both	1–4	2019	high-risk	82.04%	74.00%	90.00%
**Kenya**	Both	1–29	2019	high-risk	82.04%	74.00%	90.00%
**Kenya**	Both	1–4	2020	high-risk	61.08%	55.10%	67.01%
**Kenya**	Both	1–29	2020	high-risk	78.55%	70.86%	86.18%
**Kenya**	Both	1–4	2021	high-risk	40.49%	36.52%	44.42%
**Kenya**	Both	1–29	2021	high-risk	75.18%	67.82%	82.48%
**Mali**	Both	1–4	2010	high-risk	48.09%	39.00%	57.00%
**Mali**	Both	1–29	2010	high-risk	48.09%	39.00%	57.00%
**Mali**	Both	1–4	2011	high-risk	97.42%	94.69%	99.40%
**Mali**	Both	1–29	2011	high-risk	97.82%	95.49%	99.52%
**Mali**	Both	1–4	2012	high-risk	71.10%	69.00%	72.63%
**Mali**	Both	1–29	2012	high-risk	92.05%	89.85%	93.65%
**Mali**	Both	1–4	2013	high-risk	46.24%	44.74%	47.34%
**Mali**	Both	1–29	2013	high-risk	86.64%	84.57%	88.14%
**Mali**	Both	1–4	2014	high-risk	22.45%	21.51%	23.15%
**Mali**	Both	1–29	2014	high-risk	81.50%	79.55%	82.92%
**Mali**	Both	1–4	2015	high-risk	0.00%	0.00%	0.00%
**Mali**	Both	1–29	2015	high-risk	76.60%	74.77%	77.93%
**Mali**	Both	1–4	2016	high-risk	0.00%	0.00%	0.00%
**Mali**	Both	1–29	2016	high-risk	71.91%	70.18%	73.16%
**Mali**	Both	1–4	2017	high-risk	99.13%	97.40%	100.00%
**Mali**	Both	1–29	2017	high-risk	93.56%	91.88%	94.94%

Location	Sex	Age	Year	Analysis	Mean	Lower	Upper
**Mali**	Both	1–4	2018	high-risk	91.55%	89.68%	92.88%
**Mali**	Both	1–29	2018	high-risk	92.16%	90.49%	93.52%
**Mali**	Both	1–4	2019	high-risk	84.98%	82.84%	87.00%
**Mali**	Both	1–29	2019	high-risk	90.98%	89.35%	92.32%
**Mali**	Both	1–4	2020	high-risk	79.24%	76.46%	81.88%
**Mali**	Both	1–29	2020	high-risk	89.99%	88.47%	91.29%
**Mali**	Both	1–4	2021	high-risk	73.90%	70.62%	77.18%
**Mali**	Both	1–29	2021	high-risk	89.10%	87.63%	90.42%
**Mauritania**	Both	1–4	2010	high-risk	0.00%	0.00%	0.00%
**Mauritania**	Both	1–29	2010	high-risk	0.00%	0.00%	0.00%
**Mauritania**	Both	1–4	2011	high-risk	0.00%	0.00%	0.00%
**Mauritania**	Both	1–29	2011	high-risk	0.00%	0.00%	0.00%
**Mauritania**	Both	1–4	2012	high-risk	0.00%	0.00%	0.00%
**Mauritania**	Both	1–29	2012	high-risk	0.00%	0.00%	0.00%
**Mauritania**	Both	1–4	2013	high-risk	0.00%	0.00%	0.00%
**Mauritania**	Both	1–29	2013	high-risk	0.00%	0.00%	0.00%
**Mauritania**	Both	1–4	2014	high-risk	96.75%	93.00%	100.00%
**Mauritania**	Both	1–29	2014	high-risk	96.75%	93.00%	100.00%
**Mauritania**	Both	1–4	2015	high-risk	72.16%	69.37%	74.59%
**Mauritania**	Both	1–29	2015	high-risk	92.04%	88.47%	95.13%
**Mauritania**	Both	1–4	2016	high-risk	47.95%	46.09%	49.56%
**Mauritania**	Both	1–29	2016	high-risk	87.53%	84.13%	90.47%
**Mauritania**	Both	1–4	2017	high-risk	23.93%	23.00%	24.73%
**Mauritania**	Both	1–29	2017	high-risk	83.20%	79.97%	85.99%
**Mauritania**	Both	1–4	2018	high-risk	0.00%	0.00%	0.00%
**Mauritania**	Both	1–29	2018	high-risk	79.04%	75.97%	81.69%
**Mauritania**	Both	1–4	2019	high-risk	0.00%	0.00%	0.00%
**Mauritania**	Both	1–29	2019	high-risk	75.03%	72.12%	77.55%
**Mauritania**	Both	1–4	2020	high-risk	0.00%	0.00%	0.00%
**Mauritania**	Both	1–29	2020	high-risk	71.19%	68.43%	73.58%
**Mauritania**	Both	1–4	2021	high-risk	0.00%	0.00%	0.00%
**Mauritania**	Both	1–29	2021	high-risk	67.49%	64.88%	69.76%
**Niger**	Both	1–4	2010	high-risk	45.56%	35.00%	56.00%
**Niger**	Both	1–29	2010	high-risk	45.56%	35.00%	56.00%
**Niger**	Both	1–4	2011	high-risk	93.91%	89.63%	97.32%
**Niger**	Both	1–29	2011	high-risk	94.75%	90.89%	97.69%
**Niger**	Both	1–4	2012	high-risk	68.67%	65.39%	71.31%
**Niger**	Both	1–29	2012	high-risk	88.81%	85.19%	91.56%
**Niger**	Both	1–4	2013	high-risk	44.65%	42.30%	46.56%
**Niger**	Both	1–29	2013	high-risk	83.27%	79.88%	85.85%
**Niger**	Both	1–4	2014	high-risk	21.46%	20.07%	22.67%
**Niger**	Both	1–29	2014	high-risk	78.03%	74.84%	80.45%

Location	Sex	Age	Year	Analysis	Mean	Lower	Upper
**Niger**	Both	1–4	2015	high-risk	0.00%	0.00%	0.00%
**Niger**	Both	1–29	2015	high-risk	73.03%	70.05%	75.30%
**Niger**	Both	1–4	2016	high-risk	0.00%	0.00%	0.00%
**Niger**	Both	1–29	2016	high-risk	68.26%	65.46%	70.38%
**Niger**	Both	1–4	2017	high-risk	11.15%	10.21%	12.07%
**Niger**	Both	1–29	2017	high-risk	66.22%	63.68%	68.28%
**Niger**	Both	1–4	2018	high-risk	27.63%	25.57%	29.62%
**Niger**	Both	1–29	2018	high-risk	65.58%	63.28%	67.59%
**Niger**	Both	1–4	2019	high-risk	97.31%	94.84%	99.45%
**Niger**	Both	1–29	2019	high-risk	91.06%	88.64%	93.18%
**Niger**	Both	1–4	2020	high-risk	92.01%	90.26%	93.48%
**Niger**	Both	1–29	2020	high-risk	90.11%	87.84%	92.10%
**Niger**	Both	1–4	2021	high-risk	87.24%	85.26%	89.04%
**Niger**	Both	1–29	2021	high-risk	89.39%	87.24%	91.26%
**Nigeria**	Both	1–4	2010	high-risk	0.00%	0.00%	0.00%
**Nigeria**	Both	1–29	2010	high-risk	0.00%	0.00%	0.00%
**Nigeria**	Both	1–4	2011	high-risk	17.51%	10.98%	25.00%
**Nigeria**	Both	1–29	2011	high-risk	17.51%	10.98%	25.00%
**Nigeria**	Both	1–4	2012	high-risk	43.29%	34.75%	51.82%
**Nigeria**	Both	1–29	2012	high-risk	45.68%	36.85%	54.39%
**Nigeria**	Both	1–4	2013	high-risk	67.28%	59.68%	74.42%
**Nigeria**	Both	1–29	2013	high-risk	72.98%	65.89%	79.22%
**Nigeria**	Both	1–4	2014	high-risk	84.26%	79.00%	89.19%
**Nigeria**	Both	1–29	2014	high-risk	90.65%	86.94%	93.81%
**Nigeria**	Both	1–4	2015	high-risk	60.42%	56.01%	64.57%
**Nigeria**	Both	1–29	2015	high-risk	85.93%	82.41%	88.93%
**Nigeria**	Both	1–4	2016	high-risk	37.78%	34.34%	41.12%
**Nigeria**	Both	1–29	2016	high-risk	81.50%	78.16%	84.36%
**Nigeria**	Both	1–4	2017	high-risk	16.82%	14.48%	19.06%
**Nigeria**	Both	1–29	2017	high-risk	77.29%	74.11%	80.01%
**Nigeria**	Both	1–4	2018	high-risk	0.00%	0.00%	0.00%
**Nigeria**	Both	1–29	2018	high-risk	73.28%	70.25%	75.87%
**Nigeria**	Both	1–4	2019	high-risk	76.77%	68.55%	84.35%
**Nigeria**	Both	1–29	2019	high-risk	93.74%	90.07%	97.02%
**Nigeria**	Both	1–4	2020	high-risk	82.34%	74.63%	90.29%
**Nigeria**	Both	1–29	2020	high-risk	96.31%	92.54%	100.49%
**Nigeria**	Both	1–4	2021	high-risk	74.53%	68.89%	80.25%
**Nigeria**	Both	1–29	2021	high-risk	93.89%	90.36%	97.55%
**Senegal**	Both	1–4	2010	high-risk	0.00%	0.00%	0.00%
**Senegal**	Both	1–29	2010	high-risk	0.00%	0.00%	0.00%
**Senegal**	Both	1–4	2011	high-risk	0.00%	0.00%	0.00%
**Senegal**	Both	1–29	2011	high-risk	0.00%	0.00%	0.00%

Location	Sex	Age	Year	Analysis	Mean	Lower	Upper
**Senegal**	Both	1–4	2012	high-risk	96.18%	92.00%	99.00%
**Senegal**	Both	1–29	2012	high-risk	96.18%	92.00%	99.00%
**Senegal**	Both	1–4	2013	high-risk	71.08%	67.99%	73.17%
**Senegal**	Both	1–29	2013	high-risk	91.30%	87.34%	93.98%
**Senegal**	Both	1–4	2014	high-risk	51.60%	47.91%	55.19%
**Senegal**	Both	1–29	2014	high-risk	87.89%	84.13%	90.65%
**Senegal**	Both	1–4	2015	high-risk	27.79%	24.84%	30.97%
**Senegal**	Both	1–29	2015	high-risk	83.50%	79.93%	86.12%
**Senegal**	Both	1–4	2016	high-risk	4.37%	1.47%	7.36%
**Senegal**	Both	1–29	2016	high-risk	79.34%	75.94%	81.82%
**Senegal**	Both	1–4	2017	high-risk	2.16%	0.73%	3.65%
**Senegal**	Both	1–29	2017	high-risk	75.36%	72.14%	77.71%
**Senegal**	Both	1–4	2018	high-risk	0.00%	0.00%	0.00%
**Senegal**	Both	1–29	2018	high-risk	71.54%	68.47%	73.76%
**Senegal**	Both	1–4	2019	high-risk	0.00%	0.00%	0.00%
**Senegal**	Both	1–29	2019	high-risk	67.85%	64.93%	69.95%
**Senegal**	Both	1–4	2020	high-risk	0.00%	0.00%	0.00%
**Senegal**	Both	1–29	2020	high-risk	64.28%	61.50%	66.26%
**Senegal**	Both	1–4	2021	high-risk	0.00%	0.00%	0.00%
**Senegal**	Both	1–29	2021	high-risk	60.82%	58.17%	62.69%
**South Sudan**	Both	1–4	2010	high-risk	0.00%	0.00%	0.00%
**South Sudan**	Both	1–29	2010	high-risk	0.00%	0.00%	0.00%
**South Sudan**	Both	1–4	2011	high-risk	0.00%	0.00%	0.00%
**South Sudan**	Both	1–29	2011	high-risk	0.00%	0.00%	0.00%
**South Sudan**	Both	1–4	2012	high-risk	0.00%	0.00%	0.00%
**South Sudan**	Both	1–29	2012	high-risk	0.00%	0.00%	0.00%
**South Sudan**	Both	1–4	2013	high-risk	0.00%	0.00%	0.00%
**South Sudan**	Both	1–29	2013	high-risk	0.00%	0.00%	0.00%
**South Sudan**	Both	1–4	2014	high-risk	0.00%	0.00%	0.00%
**South Sudan**	Both	1–29	2014	high-risk	0.00%	0.00%	0.00%
**South Sudan**	Both	1–4	2015	high-risk	0.00%	0.00%	0.00%
**South Sudan**	Both	1–29	2015	high-risk	0.00%	0.00%	0.00%
**South Sudan**	Both	1–4	2016	high-risk	58.84%	50.00%	68.00%
**South Sudan**	Both	1–29	2016	high-risk	58.84%	50.00%	68.00%
**South Sudan**	Both	1–4	2017	high-risk	43.26%	36.76%	50.00%
**South Sudan**	Both	1–29	2017	high-risk	55.75%	47.38%	64.43%
**South Sudan**	Both	1–4	2018	high-risk	53.57%	43.53%	63.32%
**South Sudan**	Both	1–29	2018	high-risk	78.04%	65.58%	90.35%
**South Sudan**	Both	1–4	2019	high-risk	33.13%	26.46%	39.96%
**South Sudan**	Both	1–29	2019	high-risk	74.17%	62.52%	85.60%
**South Sudan**	Both	1–4	2020	high-risk	12.73%	8.57%	16.90%
**South Sudan**	Both	1–29	2020	high-risk	70.23%	59.20%	81.07%

Location	Sex	Age	Year	Analysis	Mean	Lower	Upper
**South Sudan**	Both	1–4	2021	high-risk	6.38%	4.28%	8.42%
**South Sudan**	Both	1–29	2021	high-risk	66.46%	56.02%	76.72%
**Sudan**	Both	1–4	2010	high-risk	0.00%	0.00%	0.00%
**Sudan**	Both	1–29	2010	high-risk	0.00%	0.00%	0.00%
**Sudan**	Both	1–4	2011	high-risk	0.00%	0.00%	0.00%
**Sudan**	Both	1–29	2011	high-risk	0.00%	0.00%	0.00%
**Sudan**	Both	1–4	2012	high-risk	46.99%	37.00%	57.02%
**Sudan**	Both	1–29	2012	high-risk	46.99%	37.00%	57.02%
**Sudan**	Both	1–4	2013	high-risk	96.04%	92.76%	98.74%
**Sudan**	Both	1–29	2013	high-risk	96.65%	93.93%	98.95%
**Sudan**	Both	1–4	2014	high-risk	71.31%	68.75%	73.43%
**Sudan**	Both	1–29	2014	high-risk	92.19%	89.59%	94.39%
**Sudan**	Both	1–4	2015	high-risk	47.04%	45.15%	48.60%
**Sudan**	Both	1–29	2015	high-risk	87.96%	85.48%	90.06%
**Sudan**	Both	1–4	2016	high-risk	99.38%	98.03%	100.00%
**Sudan**	Both	1–29	2016	high-risk	97.37%	95.28%	99.14%
**Sudan**	Both	1–4	2017	high-risk	90.85%	89.05%	92.22%
**Sudan**	Both	1–29	2017	high-risk	96.03%	94.07%	97.77%
**Sudan**	Both	1–4	2018	high-risk	83.05%	80.85%	85.14%
**Sudan**	Both	1–29	2018	high-risk	94.82%	92.87%	96.52%
**Sudan**	Both	1–4	2019	high-risk	75.71%	72.91%	78.57%
**Sudan**	Both	1–29	2019	high-risk	93.73%	91.77%	95.43%
**Sudan**	Both	1–4	2020	high-risk	68.53%	65.01%	72.24%
**Sudan**	Both	1–29	2020	high-risk	92.74%	90.87%	94.42%
**Sudan**	Both	1–4	2021	high-risk	69.71%	66.15%	73.22%
**Sudan**	Both	1–29	2021	high-risk	91.80%	89.99%	93.42%
**Togo**	Both	1–4	2010	high-risk	0.00%	0.00%	0.00%
**Togo**	Both	1–29	2010	high-risk	0.00%	0.00%	0.00%
**Togo**	Both	1–4	2011	high-risk	0.00%	0.00%	0.00%
**Togo**	Both	1–29	2011	high-risk	0.00%	0.00%	0.00%
**Togo**	Both	1–4	2012	high-risk	0.00%	0.00%	0.00%
**Togo**	Both	1–29	2012	high-risk	0.00%	0.00%	0.00%
**Togo**	Both	1–4	2013	high-risk	0.00%	0.00%	0.00%
**Togo**	Both	1–29	2013	high-risk	0.00%	0.00%	0.00%
**Togo**	Both	1–4	2014	high-risk	98.09%	95.00%	100.00%
**Togo**	Both	1–29	2014	high-risk	98.09%	95.00%	100.00%
**Togo**	Both	1–4	2015	high-risk	72.66%	70.38%	74.08%
**Togo**	Both	1–29	2015	high-risk	93.19%	90.26%	95.01%
**Togo**	Both	1–4	2016	high-risk	48.01%	46.50%	48.94%
**Togo**	Both	1–29	2016	high-risk	88.53%	85.75%	90.26%
**Togo**	Both	1–4	2017	high-risk	27.86%	24.83%	31.66%
**Togo**	Both	1–29	2017	high-risk	84.92%	82.37%	86.72%

Location	Sex	Age	Year	Analysis	Mean	Lower	Upper
**Togo**	Both	1–4	2018	high-risk	3.92%	0.74%	7.45%
**Togo**	Both	1–29	2018	high-risk	80.64%	78.22%	82.36%
**Togo**	Both	1–4	2019	high-risk	2.60%	0.49%	4.95%
**Togo**	Both	1–29	2019	high-risk	76.55%	74.24%	78.18%
**Togo**	Both	1–4	2020	high-risk	1.30%	0.25%	2.47%
**Togo**	Both	1–29	2020	high-risk	72.62%	70.44%	74.18%
**Togo**	Both	1–4	2021	high-risk	96.02%	92.00%	99.00%
**Togo**	Both	1–29	2021	high-risk	97.63%	95.38%	99.40%
**Uganda**	Both	1–4	2010	high-risk	0.00%	0.00%	0.00%
**Uganda**	Both	1–29	2010	high-risk	0.00%	0.00%	0.00%
**Uganda**	Both	1–4	2011	high-risk	0.00%	0.00%	0.00%
**Uganda**	Both	1–29	2011	high-risk	0.00%	0.00%	0.00%
**Uganda**	Both	1–4	2012	high-risk	0.00%	0.00%	0.00%
**Uganda**	Both	1–29	2012	high-risk	0.00%	0.00%	0.00%
**Uganda**	Both	1–4	2013	high-risk	0.00%	0.00%	0.00%
**Uganda**	Both	1–29	2013	high-risk	0.00%	0.00%	0.00%
**Uganda**	Both	1–4	2014	high-risk	0.00%	0.00%	0.00%
**Uganda**	Both	1–29	2014	high-risk	0.00%	0.00%	0.00%
**Uganda**	Both	1–4	2015	high-risk	0.00%	0.00%	0.00%
**Uganda**	Both	1–29	2015	high-risk	0.00%	0.00%	0.00%
**Uganda**	Both	1–4	2016	high-risk	0.00%	0.00%	0.00%
**Uganda**	Both	1–29	2016	high-risk	0.00%	0.00%	0.00%
**Uganda**	Both	1–4	2017	high-risk	89.13%	82.00%	95.00%
**Uganda**	Both	1–29	2017	high-risk	89.13%	82.00%	95.00%
**Uganda**	Both	1–4	2018	high-risk	65.94%	60.67%	70.29%
**Uganda**	Both	1–29	2018	high-risk	84.60%	77.84%	90.17%
**Uganda**	Both	1–4	2019	high-risk	43.45%	39.97%	46.31%
**Uganda**	Both	1–29	2019	high-risk	80.31%	73.89%	85.60%
**Uganda**	Both	1–4	2020	high-risk	21.50%	19.78%	22.92%
**Uganda**	Both	1–29	2020	high-risk	76.23%	70.13%	81.25%
**Uganda**	Both	1–4	2021	high-risk	0.00%	0.00%	0.00%
**Uganda**	Both	1–29	2021	high-risk	72.35%	66.56%	77.11%
**Benin**	Both	1–4	2010	full country	0.00%	0.00%	0.00%
**Benin**	Both	1–29	2010	full country	0.00%	0.00%	0.00%
**Benin**	Both	1–4	2011	full country	0.00%	0.00%	0.00%
**Benin**	Both	1–29	2011	full country	0.00%	0.00%	0.00%
**Benin**	Both	1–4	2012	full country	35.49%	26.00%	45.00%
**Benin**	Both	1–29	2012	full country	35.49%	26.00%	45.00%
**Benin**	Both	1–4	2013	full country	26.10%	19.12%	33.09%
**Benin**	Both	1–29	2013	full country	33.54%	24.57%	42.52%
**Benin**	Both	1–4	2014	full country	17.12%	12.54%	21.70%
**Benin**	Both	1–29	2014	full country	31.69%	23.22%	40.18%

Location	Sex	Age	Year	Analysis	Mean	Lower	Upper
**Benin**	Both	1–4	2015	full country	8.43%	6.17%	10.69%
**Benin**	Both	1–29	2015	full country	29.93%	21.92%	37.95%
**Benin**	Both	1–4	2016	full country	0.00%	0.00%	0.00%
**Benin**	Both	1–29	2016	full country	28.24%	20.69%	35.81%
**Benin**	Both	1–4	2017	full country	0.00%	0.00%	0.00%
**Benin**	Both	1–29	2017	full country	26.62%	19.50%	33.75%
**Benin**	Both	1–4	2018	full country	0.00%	0.00%	0.00%
**Benin**	Both	1–29	2018	full country	25.06%	18.36%	31.78%
**Benin**	Both	1–4	2019	full country	0.00%	0.00%	0.00%
**Benin**	Both	1–29	2019	full country	23.57%	17.26%	29.88%
**Benin**	Both	1–4	2020	full country	0.00%	0.00%	0.00%
**Benin**	Both	1–29	2020	full country	22.13%	16.21%	28.06%
**Benin**	Both	1–4	2021	full country	0.00%	0.00%	0.00%
**Benin**	Both	1–29	2021	full country	20.75%	15.20%	26.31%
**Burkina Faso**	Both	1–4	2010	full country	95.94%	92.00%	99.00%
**Burkina Faso**	Both	1–29	2010	full country	95.94%	92.00%	99.00%
**Burkina Faso**	Both	1–4	2011	full country	70.22%	67.33%	72.46%
**Burkina Faso**	Both	1–29	2011	full country	90.50%	86.78%	93.38%
**Burkina Faso**	Both	1–4	2012	full country	45.88%	43.99%	47.34%
**Burkina Faso**	Both	1–29	2012	full country	85.38%	81.88%	88.11%
**Burkina Faso**	Both	1–4	2013	full country	22.52%	21.59%	23.24%
**Burkina Faso**	Both	1–29	2013	full country	80.51%	77.21%	83.08%
**Burkina Faso**	Both	1–4	2014	full country	0.00%	0.00%	0.00%
**Burkina Faso**	Both	1–29	2014	full country	75.85%	72.74%	78.27%
**Burkina Faso**	Both	1–4	2015	full country	0.00%	0.00%	0.00%
**Burkina Faso**	Both	1–29	2015	full country	71.36%	68.43%	73.64%
**Burkina Faso**	Both	1–4	2016	full country	96.95%	93.00%	100.00%
**Burkina Faso**	Both	1–29	2016	full country	96.24%	93.21%	98.79%
**Burkina Faso**	Both	1–4	2017	full country	87.52%	84.70%	89.73%
**Burkina Faso**	Both	1–29	2017	full country	94.33%	91.44%	96.76%
**Burkina Faso**	Both	1–4	2018	full country	86.59%	84.62%	88.26%
**Burkina Faso**	Both	1–29	2018	full country	94.18%	91.42%	96.50%
**Burkina Faso**	Both	1–4	2019	full country	85.86%	84.33%	87.21%
**Burkina Faso**	Both	1–29	2019	full country	94.07%	91.52%	96.26%
**Burkina Faso**	Both	1–4	2020	full country	85.53%	83.99%	86.87%
**Burkina Faso**	Both	1–29	2020	full country	94.08%	91.67%	96.15%
**Burkina Faso**	Both	1–4	2021	full country	86.42%	85.09%	87.61%
**Burkina Faso**	Both	1–29	2021	full country	94.11%	91.85%	96.06%
**Burundi**	Both	1–4	2010	full country	0.00%	0.00%	0.00%
**Burundi**	Both	1–29	2010	full country	0.00%	0.00%	0.00%
**Burundi**	Both	1–4	2011	full country	0.00%	0.00%	0.00%
**Burundi**	Both	1–29	2011	full country	0.00%	0.00%	0.00%

Location	Sex	Age	Year	Analysis	Mean	Lower	Upper
**Burundi**	Both	1–4	2012	full country	0.00%	0.00%	0.00%
**Burundi**	Both	1–29	2012	full country	0.00%	0.00%	0.00%
**Burundi**	Both	1–4	2013	full country	0.00%	0.00%	0.00%
**Burundi**	Both	1–29	2013	full country	0.00%	0.00%	0.00%
**Burundi**	Both	1–4	2014	full country	0.00%	0.00%	0.00%
**Burundi**	Both	1–29	2014	full country	0.00%	0.00%	0.00%
**Burundi**	Both	1–4	2015	full country	0.00%	0.00%	0.00%
**Burundi**	Both	1–29	2015	full country	0.00%	0.00%	0.00%
**Burundi**	Both	1–4	2016	full country	0.00%	0.00%	0.00%
**Burundi**	Both	1–29	2016	full country	0.00%	0.00%	0.00%
**Burundi**	Both	1–4	2017	full country	0.00%	0.00%	0.00%
**Burundi**	Both	1–29	2017	full country	0.00%	0.00%	0.00%
**Burundi**	Both	1–4	2018	full country	99.02%	97.00%	100.00%
**Burundi**	Both	1–29	2018	full country	99.02%	97.00%	100.00%
**Burundi**	Both	1–4	2019	full country	73.46%	71.96%	74.19%
**Burundi**	Both	1–29	2019	full country	93.98%	92.06%	94.91%
**Burundi**	Both	1–4	2020	full country	48.54%	47.55%	49.02%
**Burundi**	Both	1–29	2020	full country	89.16%	87.34%	90.04%
**Burundi**	Both	1–4	2021	full country	24.09%	23.60%	24.33%
**Burundi**	Both	1–29	2021	full country	84.54%	82.82%	85.38%
**Cameroon**	Both	1–4	2010	full country	0.00%	0.00%	0.00%
**Cameroon**	Both	1–29	2010	full country	0.00%	0.00%	0.00%
**Cameroon**	Both	1–4	2011	full country	9.91%	4.00%	16.00%
**Cameroon**	Both	1–29	2011	full country	9.91%	4.00%	16.00%
**Cameroon**	Both	1–4	2012	full country	25.42%	17.48%	33.18%
**Cameroon**	Both	1–29	2012	full country	27.10%	18.77%	35.05%
**Cameroon**	Both	1–4	2013	full country	42.38%	33.90%	51.45%
**Cameroon**	Both	1–29	2013	full country	47.61%	38.67%	56.29%
**Cameroon**	Both	1–4	2014	full country	29.80%	23.66%	36.56%
**Cameroon**	Both	1–29	2014	full country	45.13%	36.66%	53.36%
**Cameroon**	Both	1–4	2015	full country	17.65%	13.62%	22.19%
**Cameroon**	Both	1–29	2015	full country	42.78%	34.74%	50.58%
**Cameroon**	Both	1–4	2016	full country	7.11%	5.06%	9.39%
**Cameroon**	Both	1–29	2016	full country	40.53%	32.91%	47.93%
**Cameroon**	Both	1–4	2017	full country	0.00%	0.00%	0.00%
**Cameroon**	Both	1–29	2017	full country	38.38%	31.16%	45.39%
**Cameroon**	Both	1–4	2018	full country	0.00%	0.00%	0.00%
**Cameroon**	Both	1–29	2018	full country	36.31%	29.48%	42.96%
**Cameroon**	Both	1–4	2019	full country	0.00%	0.00%	0.00%
**Cameroon**	Both	1–29	2019	full country	34.34%	27.88%	40.62%
**Cameroon**	Both	1–4	2020	full country	0.00%	0.00%	0.00%
**Cameroon**	Both	1–29	2020	full country	32.44%	26.34%	38.38%

Location	Sex	Age	Year	Analysis	Mean	Lower	Upper
**Cameroon**	Both	1–4	2021	full country	0.00%	0.00%	0.00%
**Cameroon**	Both	1–29	2021	full country	30.61%	24.86%	36.23%
**Central African Republic**	Both	1–4	2010	full country	0.00%	0.00%	0.00%
**Central African Republic**	Both	1–29	2010	full country	0.00%	0.00%	0.00%
**Central African Republic**	Both	1–4	2011	full country	0.00%	0.00%	0.00%
**Central African Republic**	Both	1–29	2011	full country	0.00%	0.00%	0.00%
**Central African Republic**	Both	1–4	2012	full country	0.00%	0.00%	0.00%
**Central African Republic**	Both	1–29	2012	full country	0.00%	0.00%	0.00%
**Central African Republic**	Both	1–4	2013	full country	0.00%	0.00%	0.00%
**Central African Republic**	Both	1–29	2013	full country	0.00%	0.00%	0.00%
**Central African Republic**	Both	1–4	2014	full country	0.00%	0.00%	0.00%
**Central African Republic**	Both	1–29	2014	full country	0.00%	0.00%	0.00%
**Central African Republic**	Both	1–4	2015	full country	0.00%	0.00%	0.00%
**Central African Republic**	Both	1–29	2015	full country	0.00%	0.00%	0.00%
**Central African Republic**	Both	1–4	2016	full country	0.00%	0.00%	0.00%
**Central African Republic**	Both	1–29	2016	full country	0.00%	0.00%	0.00%
**Central African Republic**	Both	1–4	2017	full country	88.83%	82.43%	94.44%
**Central African Republic**	Both	1–29	2017	full country	88.13%	81.27%	94.08%
**Central African Republic**	Both	1–4	2018	full country	77.14%	72.48%	81.47%
**Central African Republic**	Both	1–29	2018	full country	85.90%	79.49%	91.59%
**Central African Republic**	Both	1–4	2019	full country	67.27%	63.61%	70.86%
**Central African Republic**	Both	1–29	2019	full country	84.06%	78.09%	89.52%
**Central African Republic**	Both	1–4	2020	full country	59.04%	55.68%	62.68%
**Central African Republic**	Both	1–29	2020	full country	82.57%	76.87%	87.84%
**Central African Republic**	Both	1–4	2021	full country	50.72%	46.88%	54.94%
**Central African Republic**	Both	1–29	2021	full country	81.24%	75.84%	86.22%
**Chad**	Both	1–4	2010	full country	0.00%	0.00%	0.00%
**Chad**	Both	1–29	2010	full country	0.00%	0.00%	0.00%
**Chad**	Both	1–4	2011	full country	48.41%	39.00%	58.00%
**Chad**	Both	1–29	2011	full country	48.41%	39.00%	58.00%
**Chad**	Both	1–4	2012	full country	98.01%	95.61%	100.00%
**Chad**	Both	1–29	2012	full country	98.32%	96.28%	100.00%
**Chad**	Both	1–4	2013	full country	71.64%	69.76%	73.18%
**Chad**	Both	1–29	2013	full country	92.28%	90.36%	93.85%
**Chad**	Both	1–4	2014	full country	46.67%	45.28%	47.79%
**Chad**	Both	1–29	2014	full country	86.60%	84.80%	88.08%
**Chad**	Both	1–4	2015	full country	22.72%	21.80%	23.44%
**Chad**	Both	1–29	2015	full country	81.20%	79.52%	82.59%
**Chad**	Both	1–4	2016	full country	0.00%	0.00%	0.00%
**Chad**	Both	1–29	2016	full country	76.06%	74.47%	77.36%
**Chad**	Both	1–4	2017	full country	7.43%	6.59%	8.30%
**Chad**	Both	1–29	2017	full country	72.81%	71.28%	74.01%

Location	Sex	Age	Year	Analysis	Mean	Lower	Upper
**Chad**	Both	1–4	2018	full country	92.32%	86.08%	98.84%
**Chad**	Both	1–29	2018	full country	99.43%	96.57%	102.22%
**Chad**	Both	1–4	2019	full country	81.21%	76.09%	86.15%
**Chad**	Both	1–29	2019	full country	96.09%	93.40%	98.81%
**Chad**	Both	1–4	2020	full country	71.46%	67.59%	75.29%
**Chad**	Both	1–29	2020	full country	93.09%	90.50%	95.69%
**Chad**	Both	1–4	2021	full country	70.57%	63.43%	78.38%
**Chad**	Both	1–29	2021	full country	95.89%	92.23%	99.66%
**Côte d'Ivoire**	Both	1–4	2010	full country	0.00%	0.00%	0.00%
**Côte d'Ivoire**	Both	1–29	2010	full country	0.00%	0.00%	0.00%
**Côte d'Ivoire**	Both	1–4	2011	full country	0.00%	0.00%	0.00%
**Côte d'Ivoire**	Both	1–29	2011	full country	0.00%	0.00%	0.00%
**Côte d'Ivoire**	Both	1–4	2012	full country	0.00%	0.00%	0.00%
**Côte d'Ivoire**	Both	1–29	2012	full country	0.00%	0.00%	0.00%
**Côte d'Ivoire**	Both	1–4	2013	full country	0.00%	0.00%	0.00%
**Côte d'Ivoire**	Both	1–29	2013	full country	0.00%	0.00%	0.00%
**Côte d'Ivoire**	Both	1–4	2014	full country	27.40%	19.00%	36.00%
**Côte d'Ivoire**	Both	1–29	2014	full country	27.40%	19.00%	36.00%
**Côte d'Ivoire**	Both	1–4	2015	full country	20.22%	14.02%	26.57%
**Côte d'Ivoire**	Both	1–29	2015	full country	26.00%	18.02%	34.15%
**Côte d'Ivoire**	Both	1–4	2016	full country	13.30%	9.22%	17.47%
**Côte d'Ivoire**	Both	1–29	2016	full country	24.65%	17.09%	32.38%
**Côte d'Ivoire**	Both	1–4	2017	full country	6.57%	4.56%	8.63%
**Côte d'Ivoire**	Both	1–29	2017	full country	23.35%	16.19%	30.67%
**Côte d'Ivoire**	Both	1–4	2018	full country	35.45%	28.05%	43.64%
**Côte d'Ivoire**	Both	1–29	2018	full country	28.96%	21.92%	36.33%
**Côte d'Ivoire**	Both	1–4	2019	full country	44.29%	38.82%	50.63%
**Côte d'Ivoire**	Both	1–29	2019	full country	30.65%	23.93%	37.57%
**Côte d'Ivoire**	Both	1–4	2020	full country	53.97%	49.76%	58.21%
**Côte d'Ivoire**	Both	1–29	2020	full country	32.47%	26.08%	39.00%
**Côte d'Ivoire**	Both	1–4	2021	full country	64.08%	60.45%	67.64%
**Côte d'Ivoire**	Both	1–29	2021	full country	34.32%	28.24%	40.51%
**Democratic Republic of the Congo**	Both	1–4	2010	full country	0.00%	0.00%	0.00%
**Democratic Republic of the Congo**	Both	1–29	2010	full country	0.00%	0.00%	0.00%
**Democratic Republic of the Congo**	Both	1–4	2011	full country	0.00%	0.00%	0.00%
**Democratic Republic of the Congo**	Both	1–29	2011	full country	0.00%	0.00%	0.00%
**Democratic Republic of the Congo**	Both	1–4	2012	full country	0.00%	0.00%	0.00%
**Democratic Republic of the Congo**	Both	1–29	2012	full country	0.00%	0.00%	0.00%
**Democratic Republic of the Congo**	Both	1–4	2013	full country	0.00%	0.00%	0.00%
**Democratic Republic of the Congo**	Both	1–29	2013	full country	0.00%	0.00%	0.00%
**Democratic Republic of the Congo**	Both	1–4	2014	full country	0.00%	0.00%	0.00%
**Democratic Republic of the Congo**	Both	1–29	2014	full country	0.00%	0.00%	0.00%

Location	Sex	Age	Year	Analysis	Mean	Lower	Upper
**Democratic Republic of the Congo**	Both	1–4	2015	full country	0.00%	0.00%	0.00%
**Democratic Republic of the Congo**	Both	1–29	2015	full country	0.00%	0.00%	0.00%
**Democratic Republic of the Congo**	Both	1–4	2016	full country	33.58%	25.00%	44.00%
**Democratic Republic of the Congo**	Both	1–29	2016	full country	33.58%	25.00%	44.00%
**Democratic Republic of the Congo**	Both	1–4	2017	full country	24.93%	18.56%	32.66%
**Democratic Republic of the Congo**	Both	1–29	2017	full country	31.92%	23.76%	41.81%
**Democratic Republic of the Congo**	Both	1–4	2018	full country	16.49%	12.28%	21.61%
**Democratic Republic of the Congo**	Both	1–29	2018	full country	30.33%	22.58%	39.74%
**Democratic Republic of the Congo**	Both	1–4	2019	full country	8.20%	6.10%	10.74%
**Democratic Republic of the Congo**	Both	1–29	2019	full country	28.83%	21.46%	37.77%
**Democratic Republic of the Congo**	Both	1–4	2020	full country	0.00%	0.00%	0.00%
**Democratic Republic of the Congo**	Both	1–29	2020	full country	27.39%	20.39%	35.89%
**Democratic Republic of the Congo**	Both	1–4	2021	full country	0.00%	0.00%	0.00%
**Democratic Republic of the Congo**	Both	1–29	2021	full country	26.02%	19.37%	34.09%
**Eritrea**	Both	1–4	2010	full country	0.00%	0.00%	0.00%
**Eritrea**	Both	1–29	2010	full country	0.00%	0.00%	0.00%
**Eritrea**	Both	1–4	2011	full country	0.00%	0.00%	0.00%
**Eritrea**	Both	1–29	2011	full country	0.00%	0.00%	0.00%
**Eritrea**	Both	1–4	2012	full country	0.00%	0.00%	0.00%
**Eritrea**	Both	1–29	2012	full country	0.00%	0.00%	0.00%
**Eritrea**	Both	1–4	2013	full country	0.00%	0.00%	0.00%
**Eritrea**	Both	1–29	2013	full country	0.00%	0.00%	0.00%
**Eritrea**	Both	1–4	2014	full country	0.00%	0.00%	0.00%
**Eritrea**	Both	1–29	2014	full country	0.00%	0.00%	0.00%
**Eritrea**	Both	1–4	2015	full country	0.00%	0.00%	0.00%
**Eritrea**	Both	1–29	2015	full country	0.00%	0.00%	0.00%
**Eritrea**	Both	1–4	2016	full country	0.00%	0.00%	0.00%
**Eritrea**	Both	1–29	2016	full country	0.00%	0.00%	0.00%
**Eritrea**	Both	1–4	2017	full country	0.00%	0.00%	0.00%
**Eritrea**	Both	1–29	2017	full country	0.00%	0.00%	0.00%
**Eritrea**	Both	1–4	2018	full country	0.00%	0.00%	0.00%
**Eritrea**	Both	1–29	2018	full country	0.00%	0.00%	0.00%
**Eritrea**	Both	1–4	2019	full country	94.00%	89.00%	98.00%
**Eritrea**	Both	1–29	2019	full country	94.00%	89.00%	98.00%
**Eritrea**	Both	1–4	2020	full country	78.25%	74.73%	81.19%
**Eritrea**	Both	1–29	2020	full country	91.23%	86.51%	95.04%
**Eritrea**	Both	1–4	2021	full country	73.84%	71.00%	76.32%
**Eritrea**	Both	1–29	2021	full country	90.48%	85.94%	94.23%
**Ethiopia**	Both	1–4	2010	full country	0.00%	0.00%	0.00%
**Ethiopia**	Both	1–29	2010	full country	0.00%	0.00%	0.00%
**Ethiopia**	Both	1–4	2011	full country	0.00%	0.00%	0.00%
**Ethiopia**	Both	1–29	2011	full country	0.00%	0.00%	0.00%

Location	Sex	Age	Year	Analysis	Mean	Lower	Upper
**Ethiopia**	Both	1–4	2012	full country	0.00%	0.00%	0.00%
**Ethiopia**	Both	1–29	2012	full country	0.00%	0.00%	0.00%
**Ethiopia**	Both	1–4	2013	full country	33.29%	24.00%	42.00%
**Ethiopia**	Both	1–29	2013	full country	33.29%	24.00%	42.00%
**Ethiopia**	Both	1–4	2014	full country	74.85%	67.05%	82.06%
**Ethiopia**	Both	1–29	2014	full country	77.20%	69.62%	83.79%
**Ethiopia**	Both	1–4	2015	full country	99.53%	98.54%	100.00%
**Ethiopia**	Both	1–29	2015	full country	99.72%	99.11%	100.00%
**Ethiopia**	Both	1–4	2016	full country	73.80%	72.97%	74.21%
**Ethiopia**	Both	1–29	2016	full country	95.22%	94.63%	95.49%
**Ethiopia**	Both	1–4	2017	full country	48.62%	47.94%	48.96%
**Ethiopia**	Both	1–29	2017	full country	90.88%	90.31%	91.13%
**Ethiopia**	Both	1–4	2018	full country	23.97%	23.50%	24.22%
**Ethiopia**	Both	1–29	2018	full country	86.66%	86.12%	86.90%
**Ethiopia**	Both	1–4	2019	full country	0.00%	0.00%	0.00%
**Ethiopia**	Both	1–29	2019	full country	82.55%	82.03%	82.78%
**Ethiopia**	Both	1–4	2020	full country	0.00%	0.00%	0.00%
**Ethiopia**	Both	1–29	2020	full country	78.54%	78.05%	78.76%
**Ethiopia**	Both	1–4	2021	full country	0.00%	0.00%	0.00%
**Ethiopia**	Both	1–29	2021	full country	74.61%	74.14%	74.83%
**Gambia**	Both	1–4	2010	full country	0.00%	0.00%	0.00%
**Gambia**	Both	1–29	2010	full country	0.00%	0.00%	0.00%
**Gambia**	Both	1–4	2011	full country	0.00%	0.00%	0.00%
**Gambia**	Both	1–29	2011	full country	0.00%	0.00%	0.00%
**Gambia**	Both	1–4	2012	full country	0.00%	0.00%	0.00%
**Gambia**	Both	1–29	2012	full country	0.00%	0.00%	0.00%
**Gambia**	Both	1–4	2013	full country	96.63%	93.00%	100.00%
**Gambia**	Both	1–29	2013	full country	96.63%	93.00%	100.00%
**Gambia**	Both	1–4	2014	full country	71.35%	68.67%	73.84%
**Gambia**	Both	1–29	2014	full country	91.72%	88.27%	94.92%
**Gambia**	Both	1–4	2015	full country	47.01%	45.25%	48.65%
**Gambia**	Both	1–29	2015	full country	87.12%	83.85%	90.16%
**Gambia**	Both	1–4	2016	full country	23.29%	22.42%	24.11%
**Gambia**	Both	1–29	2016	full country	82.79%	79.68%	85.67%
**Gambia**	Both	1–4	2017	full country	0.00%	0.00%	0.00%
**Gambia**	Both	1–29	2017	full country	78.69%	75.73%	81.43%
**Gambia**	Both	1–4	2018	full country	0.00%	0.00%	0.00%
**Gambia**	Both	1–29	2018	full country	74.79%	71.98%	77.39%
**Gambia**	Both	1–4	2019	full country	88.38%	82.39%	93.83%
**Gambia**	Both	1–29	2019	full country	94.43%	91.45%	97.12%
**Gambia**	Both	1–4	2020	full country	81.11%	76.92%	84.86%
**Gambia**	Both	1–29	2020	full country	92.76%	89.98%	95.29%

Location	Sex	Age	Year	Analysis	Mean	Lower	Upper
**Gambia**	Both	1–4	2021	full country	81.34%	77.99%	84.35%
**Gambia**	Both	1–29	2021	full country	92.46%	89.85%	94.92%
**Ghana**	Both	1–4	2010	full country	0.00%	0.00%	0.00%
**Ghana**	Both	1–29	2010	full country	0.00%	0.00%	0.00%
**Ghana**	Both	1–4	2011	full country	0.00%	0.00%	0.00%
**Ghana**	Both	1–29	2011	full country	0.00%	0.00%	0.00%
**Ghana**	Both	1–4	2012	full country	16.17%	9.00%	24.00%
**Ghana**	Both	1–29	2012	full country	16.17%	9.00%	24.00%
**Ghana**	Both	1–4	2013	full country	11.98%	6.67%	17.78%
**Ghana**	Both	1–29	2013	full country	15.44%	8.59%	22.92%
**Ghana**	Both	1–4	2014	full country	7.91%	4.40%	11.74%
**Ghana**	Both	1–29	2014	full country	14.74%	8.20%	21.87%
**Ghana**	Both	1–4	2015	full country	3.92%	2.18%	5.82%
**Ghana**	Both	1–29	2015	full country	14.05%	7.82%	20.85%
**Ghana**	Both	1–4	2016	full country	31.32%	24.54%	38.47%
**Ghana**	Both	1–29	2016	full country	18.79%	13.01%	25.25%
**Ghana**	Both	1–4	2017	full country	43.30%	39.05%	47.49%
**Ghana**	Both	1–29	2017	full country	21.00%	15.57%	27.01%
**Ghana**	Both	1–4	2018	full country	58.52%	55.43%	61.53%
**Ghana**	Both	1–29	2018	full country	23.74%	18.66%	29.46%
**Ghana**	Both	1–4	2019	full country	68.82%	66.10%	71.24%
**Ghana**	Both	1–29	2019	full country	26.49%	21.64%	31.95%
**Ghana**	Both	1–4	2020	full country	70.73%	68.28%	72.98%
**Ghana**	Both	1–29	2020	full country	29.21%	24.60%	34.42%
**Ghana**	Both	1–4	2021	full country	72.36%	70.03%	74.50%
**Ghana**	Both	1–29	2021	full country	31.92%	27.54%	36.87%
**Guinea**	Both	1–4	2010	full country	0.00%	0.00%	0.00%
**Guinea**	Both	1–29	2010	full country	0.00%	0.00%	0.00%
**Guinea**	Both	1–4	2011	full country	0.00%	0.00%	0.00%
**Guinea**	Both	1–29	2011	full country	0.00%	0.00%	0.00%
**Guinea**	Both	1–4	2012	full country	0.00%	0.00%	0.00%
**Guinea**	Both	1–29	2012	full country	0.00%	0.00%	0.00%
**Guinea**	Both	1–4	2013	full country	0.00%	0.00%	0.00%
**Guinea**	Both	1–29	2013	full country	0.00%	0.00%	0.00%
**Guinea**	Both	1–4	2014	full country	17.72%	11.00%	25.00%
**Guinea**	Both	1–29	2014	full country	17.72%	11.00%	25.00%
**Guinea**	Both	1–4	2015	full country	44.00%	34.67%	52.99%
**Guinea**	Both	1–29	2015	full country	46.37%	37.06%	55.41%
**Guinea**	Both	1–4	2016	full country	31.84%	24.90%	38.51%
**Guinea**	Both	1–29	2016	full country	43.89%	35.08%	52.44%
**Guinea**	Both	1–4	2017	full country	20.08%	15.52%	24.45%
**Guinea**	Both	1–29	2017	full country	41.52%	33.19%	49.62%

Location	Sex	Age	Year	Analysis	Mean	Lower	Upper
**Guinea**	Both	1–4	2018	full country	8.58%	6.27%	10.86%
**Guinea**	Both	1–29	2018	full country	39.24%	31.37%	46.90%
**Guinea**	Both	1–4	2019	full country	0.00%	0.00%	0.00%
**Guinea**	Both	1–29	2019	full country	37.05%	29.61%	44.28%
**Guinea**	Both	1–4	2020	full country	0.00%	0.00%	0.00%
**Guinea**	Both	1–29	2020	full country	34.93%	27.92%	41.75%
**Guinea**	Both	1–4	2021	full country	1.32%	1.10%	1.57%
**Guinea**	Both	1–29	2021	full country	33.14%	26.52%	39.55%
**Guinea-Bissau**	Both	1–4	2010	full country	0.00%	0.00%	0.00%
**Guinea-Bissau**	Both	1–29	2010	full country	0.00%	0.00%	0.00%
**Guinea-Bissau**	Both	1–4	2011	full country	0.00%	0.00%	0.00%
**Guinea-Bissau**	Both	1–29	2011	full country	0.00%	0.00%	0.00%
**Guinea-Bissau**	Both	1–4	2012	full country	0.00%	0.00%	0.00%
**Guinea-Bissau**	Both	1–29	2012	full country	0.00%	0.00%	0.00%
**Guinea-Bissau**	Both	1–4	2013	full country	0.00%	0.00%	0.00%
**Guinea-Bissau**	Both	1–29	2013	full country	0.00%	0.00%	0.00%
**Guinea-Bissau**	Both	1–4	2014	full country	0.00%	0.00%	0.00%
**Guinea-Bissau**	Both	1–29	2014	full country	0.00%	0.00%	0.00%
**Guinea-Bissau**	Both	1–4	2015	full country	0.00%	0.00%	0.00%
**Guinea-Bissau**	Both	1–29	2015	full country	0.00%	0.00%	0.00%
**Guinea-Bissau**	Both	1–4	2016	full country	88.45%	81.00%	94.00%
**Guinea-Bissau**	Both	1–29	2016	full country	88.45%	81.00%	94.00%
**Guinea-Bissau**	Both	1–4	2017	full country	65.42%	59.91%	69.52%
**Guinea-Bissau**	Both	1–29	2017	full country	83.95%	76.88%	89.22%
**Guinea-Bissau**	Both	1–4	2018	full country	43.13%	39.50%	45.84%
**Guinea-Bissau**	Both	1–29	2018	full country	79.67%	72.96%	84.67%
**Guinea-Bissau**	Both	1–4	2019	full country	21.37%	19.57%	22.72%
**Guinea-Bissau**	Both	1–29	2019	full country	75.56%	69.20%	80.31%
**Guinea-Bissau**	Both	1–4	2020	full country	0.00%	0.00%	0.00%
**Guinea-Bissau**	Both	1–29	2020	full country	71.63%	65.60%	76.12%
**Guinea-Bissau**	Both	1–4	2021	full country	0.00%	0.00%	0.00%
**Guinea-Bissau**	Both	1–29	2021	full country	67.85%	62.13%	72.11%
**Kenya**	Both	1–4	2010	full country	0.00%	0.00%	0.00%
**Kenya**	Both	1–29	2010	full country	0.00%	0.00%	0.00%
**Kenya**	Both	1–4	2011	full country	0.00%	0.00%	0.00%
**Kenya**	Both	1–29	2011	full country	0.00%	0.00%	0.00%
**Kenya**	Both	1–4	2012	full country	0.00%	0.00%	0.00%
**Kenya**	Both	1–29	2012	full country	0.00%	0.00%	0.00%
**Kenya**	Both	1–4	2013	full country	0.00%	0.00%	0.00%
**Kenya**	Both	1–29	2013	full country	0.00%	0.00%	0.00%
**Kenya**	Both	1–4	2014	full country	0.00%	0.00%	0.00%
**Kenya**	Both	1–29	2014	full country	0.00%	0.00%	0.00%

Location	Sex	Age	Year	Analysis	Mean	Lower	Upper
**Kenya**	Both	1–4	2015	full country	0.00%	0.00%	0.00%
**Kenya**	Both	1–29	2015	full country	0.00%	0.00%	0.00%
**Kenya**	Both	1–4	2016	full country	0.00%	0.00%	0.00%
**Kenya**	Both	1–29	2016	full country	0.00%	0.00%	0.00%
**Kenya**	Both	1–4	2017	full country	0.00%	0.00%	0.00%
**Kenya**	Both	1–29	2017	full country	0.00%	0.00%	0.00%
**Kenya**	Both	1–4	2018	full country	0.00%	0.00%	0.00%
**Kenya**	Both	1–29	2018	full country	0.00%	0.00%	0.00%
**Kenya**	Both	1–4	2019	full country	7.93%	3.00%	14.00%
**Kenya**	Both	1–29	2019	full country	7.93%	3.00%	14.00%
**Kenya**	Both	1–4	2020	full country	5.91%	2.23%	10.42%
**Kenya**	Both	1–29	2020	full country	7.60%	2.87%	13.41%
**Kenya**	Both	1–4	2021	full country	3.91%	1.48%	6.91%
**Kenya**	Both	1–29	2021	full country	7.27%	2.75%	12.83%
**Mali**	Both	1–4	2010	full country	48.04%	38.00%	58.00%
**Mali**	Both	1–29	2010	full country	48.04%	38.00%	58.00%
**Mali**	Both	1–4	2011	full country	97.37%	94.38%	99.41%
**Mali**	Both	1–29	2011	full country	97.78%	95.15%	99.53%
**Mali**	Both	1–4	2012	full country	71.06%	68.77%	72.63%
**Mali**	Both	1–29	2012	full country	92.01%	89.53%	93.65%
**Mali**	Both	1–4	2013	full country	46.21%	44.51%	47.35%
**Mali**	Both	1–29	2013	full country	86.60%	84.27%	88.15%
**Mali**	Both	1–4	2014	full country	22.44%	21.28%	23.15%
**Mali**	Both	1–29	2014	full country	81.47%	79.27%	82.92%
**Mali**	Both	1–4	2015	full country	0.00%	0.00%	0.00%
**Mali**	Both	1–29	2015	full country	76.57%	74.50%	77.94%
**Mali**	Both	1–4	2016	full country	0.00%	0.00%	0.00%
**Mali**	Both	1–29	2016	full country	71.88%	69.93%	73.17%
**Mali**	Both	1–4	2017	full country	99.10%	97.38%	100.00%
**Mali**	Both	1–29	2017	full country	93.52%	91.57%	94.94%
**Mali**	Both	1–4	2018	full country	91.53%	89.69%	92.90%
**Mali**	Both	1–29	2018	full country	92.13%	90.24%	93.46%
**Mali**	Both	1–4	2019	full country	84.97%	82.85%	86.90%
**Mali**	Both	1–29	2019	full country	90.95%	89.16%	92.31%
**Mali**	Both	1–4	2020	full country	79.24%	76.54%	81.89%
**Mali**	Both	1–29	2020	full country	89.95%	88.23%	91.29%
**Mali**	Both	1–4	2021	full country	73.90%	70.62%	77.18%
**Mali**	Both	1–29	2021	full country	89.07%	87.44%	90.46%
**Mauritania**	Both	1–4	2010	full country	0.00%	0.00%	0.00%
**Mauritania**	Both	1–29	2010	full country	0.00%	0.00%	0.00%
**Mauritania**	Both	1–4	2011	full country	0.00%	0.00%	0.00%
**Mauritania**	Both	1–29	2011	full country	0.00%	0.00%	0.00%

Location	Sex	Age	Year	Analysis	Mean	Lower	Upper
**Mauritania**	Both	1–4	2012	full country	0.00%	0.00%	0.00%
**Mauritania**	Both	1–29	2012	full country	0.00%	0.00%	0.00%
**Mauritania**	Both	1–4	2013	full country	0.00%	0.00%	0.00%
**Mauritania**	Both	1–29	2013	full country	0.00%	0.00%	0.00%
**Mauritania**	Both	1–4	2014	full country	63.08%	54.00%	72.00%
**Mauritania**	Both	1–29	2014	full country	63.08%	54.00%	72.00%
**Mauritania**	Both	1–4	2015	full country	47.05%	40.28%	53.70%
**Mauritania**	Both	1–29	2015	full country	60.01%	51.37%	68.49%
**Mauritania**	Both	1–4	2016	full country	31.26%	26.76%	35.68%
**Mauritania**	Both	1–29	2016	full country	57.07%	48.85%	65.14%
**Mauritania**	Both	1–4	2017	full country	15.60%	13.36%	17.81%
**Mauritania**	Both	1–29	2017	full country	54.24%	46.44%	61.91%
**Mauritania**	Both	1–4	2018	full country	0.00%	0.00%	0.00%
**Mauritania**	Both	1–29	2018	full country	51.53%	44.11%	58.82%
**Mauritania**	Both	1–4	2019	full country	0.00%	0.00%	0.00%
**Mauritania**	Both	1–29	2019	full country	48.92%	41.88%	55.84%
**Mauritania**	Both	1–4	2020	full country	0.00%	0.00%	0.00%
**Mauritania**	Both	1–29	2020	full country	46.41%	39.73%	52.97%
**Mauritania**	Both	1–4	2021	full country	0.00%	0.00%	0.00%
**Mauritania**	Both	1–29	2021	full country	44.00%	37.67%	50.23%
**Niger**	Both	1–4	2010	full country	45.46%	36.00%	55.02%
**Niger**	Both	1–29	2010	full country	45.46%	36.00%	55.02%
**Niger**	Both	1–4	2011	full country	93.91%	89.98%	97.29%
**Niger**	Both	1–29	2011	full country	94.75%	91.27%	97.72%
**Niger**	Both	1–4	2012	full country	68.67%	65.63%	71.29%
**Niger**	Both	1–29	2012	full country	88.81%	85.55%	91.59%
**Niger**	Both	1–4	2013	full country	44.64%	42.55%	46.55%
**Niger**	Both	1–29	2013	full country	83.27%	80.20%	85.88%
**Niger**	Both	1–4	2014	full country	21.46%	20.07%	22.67%
**Niger**	Both	1–29	2014	full country	78.02%	75.15%	80.47%
**Niger**	Both	1–4	2015	full country	0.00%	0.00%	0.00%
**Niger**	Both	1–29	2015	full country	73.03%	70.33%	75.32%
**Niger**	Both	1–4	2016	full country	0.00%	0.00%	0.00%
**Niger**	Both	1–29	2016	full country	68.25%	65.72%	70.40%
**Niger**	Both	1–4	2017	full country	11.15%	10.21%	12.07%
**Niger**	Both	1–29	2017	full country	66.22%	63.89%	68.26%
**Niger**	Both	1–4	2018	full country	27.63%	25.57%	29.62%
**Niger**	Both	1–29	2018	full country	65.57%	63.29%	67.55%
**Niger**	Both	1–4	2019	full country	97.27%	94.67%	99.45%
**Niger**	Both	1–29	2019	full country	91.04%	88.73%	93.12%
**Niger**	Both	1–4	2020	full country	91.99%	90.16%	93.39%
**Niger**	Both	1–29	2020	full country	90.09%	87.94%	92.06%

Location	Sex	Age	Year	Analysis	Mean	Lower	Upper
**Niger**	Both	1–4	2021	full country	87.23%	85.29%	89.05%
**Niger**	Both	1–29	2021	full country	89.37%	87.29%	91.27%
**Nigeria**	Both	1–4	2010	full country	0.00%	0.00%	0.00%
**Nigeria**	Both	1–29	2010	full country	0.00%	0.00%	0.00%
**Nigeria**	Both	1–4	2011	full country	11.26%	5.00%	18.00%
**Nigeria**	Both	1–29	2011	full country	11.26%	5.00%	18.00%
**Nigeria**	Both	1–4	2012	full country	29.15%	20.95%	38.00%
**Nigeria**	Both	1–29	2012	full country	30.98%	22.49%	39.81%
**Nigeria**	Both	1–4	2013	full country	48.06%	39.55%	56.19%
**Nigeria**	Both	1–29	2013	full country	53.54%	44.91%	61.93%
**Nigeria**	Both	1–4	2014	full country	64.05%	56.42%	71.38%
**Nigeria**	Both	1–29	2014	full country	73.18%	66.45%	79.81%
**Nigeria**	Both	1–4	2015	full country	44.71%	38.41%	50.43%
**Nigeria**	Both	1–29	2015	full country	69.37%	62.98%	75.65%
**Nigeria**	Both	1–4	2016	full country	26.70%	22.37%	30.80%
**Nigeria**	Both	1–29	2016	full country	65.78%	59.72%	71.74%
**Nigeria**	Both	1–4	2017	full country	11.00%	8.45%	13.51%
**Nigeria**	Both	1–29	2017	full country	62.36%	56.61%	68.02%
**Nigeria**	Both	1–4	2018	full country	0.00%	0.00%	0.00%
**Nigeria**	Both	1–29	2018	full country	59.10%	53.65%	64.47%
**Nigeria**	Both	1–4	2019	full country	52.89%	44.03%	61.56%
**Nigeria**	Both	1–29	2019	full country	72.36%	66.14%	77.82%
**Nigeria**	Both	1–4	2020	full country	60.99%	52.98%	68.76%
**Nigeria**	Both	1–29	2020	full country	74.27%	68.63%	80.00%
**Nigeria**	Both	1–4	2021	full country	60.88%	55.06%	66.53%
**Nigeria**	Both	1–29	2021	full country	73.66%	68.44%	79.03%
**Senegal**	Both	1–4	2010	full country	0.00%	0.00%	0.00%
**Senegal**	Both	1–29	2010	full country	0.00%	0.00%	0.00%
**Senegal**	Both	1–4	2011	full country	0.00%	0.00%	0.00%
**Senegal**	Both	1–29	2011	full country	0.00%	0.00%	0.00%
**Senegal**	Both	1–4	2012	full country	47.87%	38.00%	57.00%
**Senegal**	Both	1–29	2012	full country	47.87%	38.00%	57.00%
**Senegal**	Both	1–4	2013	full country	35.38%	28.08%	42.13%
**Senegal**	Both	1–29	2013	full country	45.44%	36.07%	54.11%
**Senegal**	Both	1–4	2014	full country	26.80%	21.44%	32.32%
**Senegal**	Both	1–29	2014	full country	45.74%	37.11%	54.20%
**Senegal**	Both	1–4	2015	full country	14.41%	11.37%	17.86%
**Senegal**	Both	1–29	2015	full country	43.45%	35.25%	51.49%
**Senegal**	Both	1–4	2016	full country	2.22%	0.49%	4.41%
**Senegal**	Both	1–29	2016	full country	41.28%	33.49%	48.92%
**Senegal**	Both	1–4	2017	full country	1.10%	0.24%	2.19%
**Senegal**	Both	1–29	2017	full country	39.22%	31.81%	46.47%

Location	Sex	Age	Year	Analysis	Mean	Lower	Upper
**Senegal**	Both	1–4	2018	full country	0.00%	0.00%	0.00%
**Senegal**	Both	1–29	2018	full country	37.23%	30.20%	44.11%
**Senegal**	Both	1–4	2019	full country	0.00%	0.00%	0.00%
**Senegal**	Both	1–29	2019	full country	35.31%	28.64%	41.83%
**Senegal**	Both	1–4	2020	full country	0.00%	0.00%	0.00%
**Senegal**	Both	1–29	2020	full country	33.45%	27.13%	39.63%
**Senegal**	Both	1–4	2021	full country	0.00%	0.00%	0.00%
**Senegal**	Both	1–29	2021	full country	31.65%	25.67%	37.50%
**South Sudan**	Both	1–4	2010	full country	0.00%	0.00%	0.00%
**South Sudan**	Both	1–29	2010	full country	0.00%	0.00%	0.00%
**South Sudan**	Both	1–4	2011	full country	0.00%	0.00%	0.00%
**South Sudan**	Both	1–29	2011	full country	0.00%	0.00%	0.00%
**South Sudan**	Both	1–4	2012	full country	0.00%	0.00%	0.00%
**South Sudan**	Both	1–29	2012	full country	0.00%	0.00%	0.00%
**South Sudan**	Both	1–4	2013	full country	0.00%	0.00%	0.00%
**South Sudan**	Both	1–29	2013	full country	0.00%	0.00%	0.00%
**South Sudan**	Both	1–4	2014	full country	0.00%	0.00%	0.00%
**South Sudan**	Both	1–29	2014	full country	0.00%	0.00%	0.00%
**South Sudan**	Both	1–4	2015	full country	0.00%	0.00%	0.00%
**South Sudan**	Both	1–29	2015	full country	0.00%	0.00%	0.00%
**South Sudan**	Both	1–4	2016	full country	58.70%	49.98%	68.02%
**South Sudan**	Both	1–29	2016	full country	58.70%	49.98%	68.02%
**South Sudan**	Both	1–4	2017	full country	43.16%	36.75%	50.02%
**South Sudan**	Both	1–29	2017	full country	55.62%	47.35%	64.45%
**South Sudan**	Both	1–4	2018	full country	53.74%	44.94%	63.36%
**South Sudan**	Both	1–29	2018	full country	78.16%	66.78%	90.53%
**South Sudan**	Both	1–4	2019	full country	33.28%	27.24%	39.97%
**South Sudan**	Both	1–29	2019	full country	74.28%	63.63%	85.89%
**South Sudan**	Both	1–4	2020	full country	12.85%	9.17%	17.47%
**South Sudan**	Both	1–29	2020	full country	70.34%	60.26%	81.34%
**South Sudan**	Both	1–4	2021	full country	6.44%	4.52%	8.73%
**South Sudan**	Both	1–29	2021	full country	66.57%	57.02%	76.97%
**Sudan**	Both	1–4	2010	full country	0.00%	0.00%	0.00%
**Sudan**	Both	1–29	2010	full country	0.00%	0.00%	0.00%
**Sudan**	Both	1–4	2011	full country	0.00%	0.00%	0.00%
**Sudan**	Both	1–29	2011	full country	0.00%	0.00%	0.00%
**Sudan**	Both	1–4	2012	full country	47.04%	37.00%	57.00%
**Sudan**	Both	1–29	2012	full country	47.04%	37.00%	57.00%
**Sudan**	Both	1–4	2013	full country	96.09%	92.90%	98.74%
**Sudan**	Both	1–29	2013	full country	96.68%	93.93%	98.95%
**Sudan**	Both	1–4	2014	full country	71.34%	68.84%	73.43%
**Sudan**	Both	1–29	2014	full country	92.22%	89.59%	94.39%

Location	Sex	Age	Year	Analysis	Mean	Lower	Upper
**Sudan**	Both	1–4	2015	full country	47.06%	45.21%	48.60%
**Sudan**	Both	1–29	2015	full country	87.99%	85.48%	90.06%
**Sudan**	Both	1–4	2016	full country	99.33%	98.02%	100.00%
**Sudan**	Both	1–29	2016	full country	97.39%	95.28%	99.14%
**Sudan**	Both	1–4	2017	full country	90.80%	89.00%	92.17%
**Sudan**	Both	1–29	2017	full country	96.04%	94.06%	97.81%
**Sudan**	Both	1–4	2018	full country	83.02%	80.74%	85.08%
**Sudan**	Both	1–29	2018	full country	94.83%	92.89%	96.50%
**Sudan**	Both	1–4	2019	full country	75.70%	72.81%	78.53%
**Sudan**	Both	1–29	2019	full country	93.75%	91.88%	95.41%
**Sudan**	Both	1–4	2020	full country	68.53%	65.01%	72.24%
**Sudan**	Both	1–29	2020	full country	92.75%	90.88%	94.37%
**Sudan**	Both	1–4	2021	full country	69.71%	66.15%	73.22%
**Sudan**	Both	1–29	2021	full country	91.81%	90.02%	93.42%
**Togo**	Both	1–4	2010	full country	0.00%	0.00%	0.00%
**Togo**	Both	1–29	2010	full country	0.00%	0.00%	0.00%
**Togo**	Both	1–4	2011	full country	0.00%	0.00%	0.00%
**Togo**	Both	1–29	2011	full country	0.00%	0.00%	0.00%
**Togo**	Both	1–4	2012	full country	0.00%	0.00%	0.00%
**Togo**	Both	1–29	2012	full country	0.00%	0.00%	0.00%
**Togo**	Both	1–4	2013	full country	0.00%	0.00%	0.00%
**Togo**	Both	1–29	2013	full country	0.00%	0.00%	0.00%
**Togo**	Both	1–4	2014	full country	58.11%	48.00%	67.00%
**Togo**	Both	1–29	2014	full country	58.11%	48.00%	67.00%
**Togo**	Both	1–4	2015	full country	43.05%	35.56%	49.63%
**Togo**	Both	1–29	2015	full country	55.21%	45.60%	63.65%
**Togo**	Both	1–4	2016	full country	28.44%	23.49%	32.79%
**Togo**	Both	1–29	2016	full country	52.45%	43.32%	60.47%
**Togo**	Both	1–4	2017	full country	16.77%	13.37%	20.57%
**Togo**	Both	1–29	2017	full country	51.36%	43.16%	58.71%
**Togo**	Both	1–4	2018	full country	2.29%	0.00%	5.21%
**Togo**	Both	1–29	2018	full country	48.77%	40.99%	55.74%
**Togo**	Both	1–4	2019	full country	1.52%	0.00%	3.46%
**Togo**	Both	1–29	2019	full country	46.29%	38.90%	52.90%
**Togo**	Both	1–4	2020	full country	0.76%	0.00%	1.73%
**Togo**	Both	1–29	2020	full country	43.92%	36.91%	50.18%
**Togo**	Both	1–4	2021	full country	69.91%	61.00%	79.00%
**Togo**	Both	1–29	2021	full country	63.91%	57.13%	70.32%
**Uganda**	Both	1–4	2010	full country	0.00%	0.00%	0.00%
**Uganda**	Both	1–29	2010	full country	0.00%	0.00%	0.00%
**Uganda**	Both	1–4	2011	full country	0.00%	0.00%	0.00%
**Uganda**	Both	1–29	2011	full country	0.00%	0.00%	0.00%

Location	Sex	Age	Year	Analysis	Mean	Lower	Upper
**Uganda**	Both	1–4	2012	full country	0.00%	0.00%	0.00%
**Uganda**	Both	1–29	2012	full country	0.00%	0.00%	0.00%
**Uganda**	Both	1–4	2013	full country	0.00%	0.00%	0.00%
**Uganda**	Both	1–29	2013	full country	0.00%	0.00%	0.00%
**Uganda**	Both	1–4	2014	full country	0.00%	0.00%	0.00%
**Uganda**	Both	1–29	2014	full country	0.00%	0.00%	0.00%
**Uganda**	Both	1–4	2015	full country	0.00%	0.00%	0.00%
**Uganda**	Both	1–29	2015	full country	0.00%	0.00%	0.00%
**Uganda**	Both	1–4	2016	full country	0.00%	0.00%	0.00%
**Uganda**	Both	1–29	2016	full country	0.00%	0.00%	0.00%
**Uganda**	Both	1–4	2017	full country	21.74%	14.00%	31.00%
**Uganda**	Both	1–29	2017	full country	21.74%	14.00%	31.00%
**Uganda**	Both	1–4	2018	full country	16.09%	10.36%	22.94%
**Uganda**	Both	1–29	2018	full country	20.64%	13.29%	29.43%
**Uganda**	Both	1–4	2019	full country	10.60%	6.82%	15.11%
**Uganda**	Both	1–29	2019	full country	19.59%	12.61%	27.93%
**Uganda**	Both	1–4	2020	full country	5.25%	3.38%	7.48%
**Uganda**	Both	1–29	2020	full country	18.60%	11.97%	26.51%
**Uganda**	Both	1–4	2021	full country	0.00%	0.00%	0.00%
**Uganda**	Both	1–29	2021	full country	17.65%	11.36%	25.16%

Table S4: Coverage estimates for high-risk and full country populations for the meningitis belt, for 1–29 and 1–4 age groups, from 2010 to 2021. Year-end coverage values shown for each year. This information is also provided on the GHDx, at https://ghdx.healthdata.org/record/ihme-data/sub-saharan-africa-menafrivac-estimates-2010-2021

References

1 World Health Organization = Organisation mondiale de la Santé. Control of epidemic meningitis in countries in the African meningitis belt, 2020 – Lutte contre la méningite épidémique dans les pays de la ceinture africaine de la méningite, 2020. *Weekly Epidemiological Record = Relevé épidémiologique hebdomadaire* 2021; **96**: 365–75.

2 Bwaka A, Bita A, Lingani C, *et al.* Status of the Rollout of the Meningococcal Serogroup A Conjugate Vaccine in African Meningitis Belt Countries in 2018. *J Infect Dis* 2019; **220**: S140–7.

3 World Health Organization = Organisation mondiale de la Santé. Control of epidemic meningitis in countries in the African meningitis belt, 2019 – Lutte contre la méningite épidémique dans les pays de la ceinture africaine de la méningite, 2019. *Weekly Epidemiological Record = Relevé épidémiologique hebdomadaire* 2020; **95**: 133–43.

4 Immunization coverage cluster survey : reference manual. 2005. https://apps.who.int/iris/handle/10665/69087 (accessed Sept 7, 2022).

5 Danovaro-Holliday MC, Dansereau E, Rhoda DA, Brown DW, Cutts FT, Gacic-Dobo M. Collecting and using reliable vaccination coverage survey estimates: Summary and recommendations from the "Meeting to share lessons learnt from the roll-out of the updated WHO Vaccination Coverage Cluster Survey Reference Manual and to set an operational research agenda around vaccination coverage surveys", Geneva, 18–21 April 2017. *Vaccine* 2018; **36**: 5150–9.

6 Djingarey MH, Diomandé FVK, Barry R, *et al.* Introduction and Rollout of a New Group A Meningococcal Conjugate Vaccine (PsA-TT) in African Meningitis Belt Countries, 2010-2014. *Clin Infect Dis* 2015; **61 Suppl 5**: S434-441.

7 Galles NC, Liu PY, Updike RL, *et al.* Measuring routine childhood vaccination coverage in 204 countries and territories, 1980–2019: a systematic analysis for the Global Burden of Disease Study 2020, Release 1. *The Lancet* 2021; **398**: 503–21.
