# Supplementary material for: Meningococcal A conjugate vaccine coverage in the meningitis belt of Africa from 2010 to 2021: a modelling study
Source: eClinicalMedicine. 2023 Jan 5;56:101797. doi: 10.1016/j.eclinm.2022.101797 (PMC9985031; doi:10.1016/j.eclinm.2022.101797)
Supplement: Caption for Supplementary Material [file mmc2.docx]

**Captions for supplementary tables and figures**

Table S1: Estimated proportion of national population living in high-risk regions, by country.

Figure S1: Survey:administrative coefficients used to adjust administrative for select vaccines up to the level of survey. Coefficients are sourced from the Global Burden of Disease study, for country-years with routine infant MenA immunisation in the meningitis belt. Points show the single vaccine adjustment coefficient for each vaccine, and the black line shows the average of the four coefficients. The coefficient is defined as survey:administrative, so this coefficient is multiplied by administrative data points to produce a value adjusted to the level of survey.

Figure S2: Comparison of survey:administrative bias adjustment coefficients for single vaccines to the averaged coefficient for 2021, which is used in routine MenA vaccine coverage adjustment.

Figure S3: Comparison of country official vs. admin data reported by countries to the WHO immunisation data portal through the WHO/UNICEF Joint Reporting Form on Immunization (JRF) from 2016 to 2021 (a), adjusted admin to admin reported via JRF (b), and adjusted admin to country official reported via JRF (c).

Table S2: Bias adjustment factors for campaign MenA vaccine coverage.

Table S3: Checklist of information from GATHER included in the current report.

Figure S4: Relationship between the natural log of pre-MenAfriVac (year 2009) mortality rate in children under 5 due to meningococcal meningitis and peak MenAfriVac coverage in high-risk populations for ages 1-4.

Figure S5: Coverage estimates for full country populations for the meningitis belt, at year end 2021. (a) shows the routine immunisation values for the target age, which varies by country from 9 to 18 months. (b) shows combined coverage estimates for mass campaigns, catch-up campaigns, and routine immunisation coverage combined for ages 1 to 4. (c) shows combined coverage estimates for ages 1 to 29. Analogous to main text figure 3.

Figure S6: Coverage estimates for full country populations for the meningitis belt, for 1 to 29 and 1 to 4 age groups, from 2010 to 2021. Year-end coverage values shown for each year. Analogous to main text figure 4.

Table S4: Coverage estimates for high-risk and full country populations for the meningitis belt, for 1 to 29 and 1 to 4 age groups, from 2010 to 2021. Year-end coverage values shown for each year. This information is also provided on the GHDx, at <https://ghdx.healthdata.org/record/ihme-data/sub-saharan-africa-menafrivac-estimates-2010-2021>.
